# Harnessing the power of ferulic acid as a dual-action strategy against biofilm-forming extensively drug-resistant *Acinetobacter baumannii* isolated from cancer patients: in vitro and in vivo study

**DOI:** 10.1186/s12866-026-05006-7

**Published:** 2026-04-13

**Authors:** Noha A. Attia, Fatma I. Sonbol, Amal M. Abo-Kamar, Lamiaa A. Al-Madboly, Ahmed A. Abdelaziz

**Affiliations:** Department of Microbiology and Immunology, Faculty of Pharmacy, Tanta University, Tanta, Egypt

**Keywords:** *Acinetobacter baumannii*, Extensively Drug-Resistant, Biofilm, Ferulic acid

## Abstract

The increasing prevalence of extensively drug-resistant *Acinetobacter baumannii* (XDRAb), particularly biofilm-producing strains, poses a significant threat to clinical management, especially in immunocompromised patients. This study investigates the potential of ferulic acid (FA) as a dual-function therapeutic agent with antibacterial and antibiofilm capabilities. A total of 34 XDRAb isolates were obtained from cancer patients, and FA’s activity was assessed using a range of in vitro assays and in vivo neutropenic rat wound infection model. FA exhibited marked antimicrobial activity against XDRAb (MIC: 512 to 1024 µg/mL). At su-binhibitory concentrations (¼ and ½ MIC), FA significantly inhibited biofilm formation by 43.2 and 67.6% and disrupted established biofilms by 29.4 and 54.8% (*p* < 0.05), respectively. Consistent with these effects, FA also reduced cell surface hydrophobicity and exopolysaccharide production in XDRAb isolates. Microscopic imaging via light, scanning electron, and confocal laser scanning microscopy corroborated these findings, revealing substantial structural degradation of biofilms upon treatment. At the molecular level, FA significantly downregulated (*p* < 0.05) the key biofilm-associated genes (*abaI*, *bfmR*, *bap*, *csuE*, and *pgaB*), as quantified by RT-qPCR. In vivo, FA significantly enhanced the wound healing rates (approximately 82%, *p* < 0.05) and reduced the wound bacterial burden (*p* < 0.05). Histopathological examination showed near-complete restoration of epithelium architecture seven days post-treatment in the FA-treated group. Collectively, this study demonstrates that FA exerts combined antimicrobial and antibiofilm activities against XDRAb in preclinical models. Our findings indicate that FA interferes with multiple stages of biofilm development while exhibiting antibacterial activity at higher concentrations. These results support further investigation of FA as antibiofilm or adjunct candidate, particularly for localized biofilm-associated XDRAb infections.

## Introduction

*Acinetobacter baumannii* has emerged as a major cause of hospital-acquired infections and represents a major global health concern due to its remarkable capacity to acquire resistance to multiple antibiotic classes [1]. The World Health Organization has listed carbapenem-resistant *A. baumannii* among the highest priority pathogens within the ESKAPE group, reflecting its disproportionate contribution to multidrug-resistant infections in healthcare settings [2]. Infection risk is amplified in hospitalized patients by prolonged admission, invasive procedures, and comorbidities, and is particularly pronounced among cancer patients, where immunosuppression related to malignancy and anticancer therapy, intensive antimicrobial exposure, mucosal barrier injury, recurrent admissions, and the use of invasive devices markedly increase susceptibility to colonization and infection by resistant organisms [3, 4]. Consistent with its “red alert” status, *A. baumannii* frequently exhibits multidrug resistance and may progress to extensive drug resistance, severely limiting therapeutic options [5]. The growing prevalence of extensively drug-resistant *A. baumannii* (XDRAb), including strains with reduced susceptibility to last-resort agents, poses a serious challenge to infection control and patient outcomes, particularly in immunocompromised populations [6].

Beyond conventional resistance mechanisms, the ability of *A. baumannii* to form robust biofilms on biotic and abiotic surfaces is a key determinant of its persistence and treatment failure. Biofilm formation enhances tolerance to antimicrobial agents, facilitates environmental survival, and promotes chronic and recurrent infections in healthcare settings [7]. Consequently, infections associated with biofilm-producing XDRAb are often refractory to standard antimicrobial therapy, underscoring the need for alternative strategies that target biofilm formation and maintenance rather than bacterial viability alone [8]. In response to this challenge, nontraditional antimicrobial approaches are increasingly explored, including plant-derived secondary metabolites, antimicrobial peptides, bacteriophages, and probiotics [9].

Among these, phenolic compounds such as cinnamic acid derivatives have attracted attention due to their ability to increase membrane permeability, induce leakage of cytoplasmic contents, and alter cell morphology. Moreover, they disrupt biofilm architecture and interfere with quorum sensing pathways, often at sub-inhibitory concentrations [10–12]. Ferulic acid (FA), a naturally occurring hydroxycinnamic acid, has demonstrated antioxidant, anti-inflammatory, antibacterial, and anticancer properties, making it a promising candidate for drug development [13, 14].

Despite these findings, the potential of FA as a dual-action agent targeting both planktonic growth and biofilm-associated virulence in XDRAb, particularly isolates derived from cancer patients, remains insufficiently characterized. Moreover, data integrating phenotypic antibiofilm activity with in vivo efficacy and molecular mechanisms are limited. To address this gap, the present study systematically evaluates the antibacterial and antibiofilm effects of FA against clinical XDRAb isolates recovered from cancer patients. We assessed its impact on bacterial growth, biofilm formation, disruption of established biofilms, and exopolysaccharides production, alongside molecular analysis of quorum sensing and biofilm-related gene expression. In addition, in vivo efficacy was examined using a cyclophosphamide-induced neutropenic rat wound infection model, providing translational relevance in an immunocompromised host. By targeting biofilm integrity alongside antibacterial activity, this work explores a resistance-sparing strategy for managing hard-to-treat XDRAb infections.

## Methods

### Bacterial isolates

In this study, 100 clinical isolates of *A. baumannii* were collected between August 2020 and 2022 from hospitalized patients at the Oncology Departments of Tanta University Hospitals, Egypt. The isolates were obtained from a range of clinical specimens, including sputum, endotracheal aspirates, surgical wound exudates, abscesses, and blood samples. Preliminary identification at the genus level was performed using conventional phenotypic methods. Confirmation of species identity was achieved via molecular detection of *bla*_OXA−51−like_ gene, which is intrinsically associated with *A. baumannii*, as previously described [15]. The strain *A. baumannii* ATCC 19,606 served as a positive control. All clinical isolates were stored at − 80 °C in nutrient broth containing 20% glycerol for further analyses [16].

### Antimicrobial Susceptibility Testing (AST)

Antimicrobial susceptibility testing of the *A. baumannii* isolates was conducted following the Clinical and Laboratory Standards Institute (CLSI, 2020) guidelines [17]. The susceptibility profiles were evaluated against twenty antimicrobial agents representing nine distinct antibiotic classes. The Kirby–Bauer disk diffusion technique was employed on Mueller-Hinton agar (MHA) plates (Hi-Media, India) to assess the isolates’ response to these agents: piperacillin (PRL, 100 µg), piperacillin-tazobactam (TZP, 100/10 µg), ampicillin-sulbactam (SAM, 10/10 µg), cefepime (FEP, 30 µg), ceftazidime (CAZ, 30 µg), ceftriaxone (CRO, 30 µg), cefotaxime (CTX, 30 µg), imipenem (IPM, 10 µg), meropenem (MRP, 10 µg), doripenem (DRP, 10 µg), gentamicin (CN, 10 µg), tobramycin (TOB, 10 µg), amikacin (AK, 30 µg), tetracycline (TE, 30 µg), doxycycline (DO, 30 µg), ciprofloxacin (CIP, 5 µg), levofloxacin (LEV, 5 µg), gatifloxacin (GAT, 5 µg), trimethoprim/sulfamethoxazole (SXT, 1.25/23.75 µg) (Oxoid, England), while Colistin (CT) (Sigma-Aldrich, United States) susceptibility was evaluated separately using the broth microdilution method. After a 24-h incubation at 37 °C, 30 µL of 0.015% (w/v) resazurin solution (Sigma-Aldrich, United States) was added to each well of the 96-well microplate, followed by an additional 2-h incubation. A color shift of resazurin from blue to pink was interpreted as a positive indicator of bacterial growth [18]. *Escherichia coli* ATCC 25,922 was employed as a quality control reference strain to validate the antimicrobial susceptibility testing procedures. Interpretation of susceptibility results was carried out following the CLSI, 2020 protocol.

According to [5], isolates that resist ≥ 3 classes of antimicrobials were categorized as MDR, while isolates demonstrating resistance to all but two or fewer classes were labelled XDR. All the antimicrobial groups assigned by Magiorakos et al.. were used in this work. The multiple antibiotic resistance (MAR) index is a metric that indicates the dissemination of resistant microorganisms within a specific population. The MAR index values for each isolate were calculated using the following formulas [19] :

$$\begin{aligned}&MAR\;index\:for\:isolates\\&=\frac{Number\:of\:antibiotics\:to\:which\:the\:isolate\:was\:resistant}{Total\:number\:of\:antibiotics\:to\:which\:the\:isolate\:was\:exposed}\end{aligned}$$

MAR index values exceeding 0.2 indicate that the bacterial isolates likely originated from environments with high antibiotic exposure.

### Screening for biofilm formation by microtiter plate assay

The biofilm-forming capability of Ab isolates was estimated using the microtiter plate method [20]. Overnight cultures in Tryptic Soy Broth (TSB; Oxoid, UK) were adjusted to a 0.5 McFarland standard. Each well of 96-well flat-bottom microtiter plates received 180 µL of 1% glucose-supplemented TSB, inoculated with 20 µL of standardized bacterial suspension, followed by static incubation at 37 °C for 48 h to facilitate biofilm formation. Post incubation, biofilms were washed twice with phosphate-buffered saline (PBS) to remove non-adherent cells, fixed by air-drying, and stained with 0.1% crystal violet for 15 min. Following crystal violet staining, unbound dye was removed by thorough washing with deionized water. Biofilm-incorporated crystal violet was then solubilized using 33% (v/v) glacial acetic acid for 15 min. The optical density (OD) of the solubilized dye was measured at 595 nm using a microplate reader (Sunrise™, TECAN, Switzerland). All assays were conducted in triplicate. The reference strain *A. baumannii* ATCC 19,606 served as the positive control, while sterile TSB supplemented with glucose was used as the negative control. The cutoff value (ODc) was defined as the mean OD of the negative control plus three standard deviations (ODc = mean ODnegative + 3 × SD). Based on this ODc, isolates were categorized into four distinct phenotypic groups: strong biofilm formation (4 × ODc < OD), moderate biofilm formation (2 × ODc < OD ≤ 4 × ODc), weak biofilm formation (ODc < OD ≤ 2 × ODc), and no biofilm formation (OD ≤ ODc).

### Antibacterial activities of ferulic acid

A working solution of FA (Sigma-Aldrich, United States) was prepared in 0.5% dimethylsulfoxide (DMSO) just prior to use, and filter-sterilized using a 0.22-µm-pore-size filter. Subsequently, 100 µL of cation-adjusted Mueller-Hinton broth (CAMHB; Hi-Media, India) was pipetted into each well of a sterile 96-well microtiter plate. Two-fold serial dilutions of FA were then prepared in the broth to achieve a final volume of 100 µL per well. Each well received a 100 µL aliquot of an overnight culture of the test isolates adjusted to the standardized inoculum (1–2 × 10⁶ CFU/mL) in CAMHB. The plates were incubated at 37 °C for 18–24 h [21]. Positive controls included bacterial cultures in CAMHB supplemented with 0.5% DMSO but lacking FA, whereas negative controls consisted of uninoculated wells. The MIC of FA was defined as the lowest concentration at which no visible bacterial growth was observed, indicated by the maintenance of the blue color of the resazurin dye [18].

### Effect of sub-inhibitory concentrations of ferulic acid on growth rate of tested isolates

Growth kinetics of five representative isolates, each from strong, moderate, and weak biofilm producers, along with the ATCC 19606 strain, were monitored. Each well of 96-well microtiter plates received 180 µL of TSB, inoculated with 20 µL of standardized bacterial suspension (0.5 McFarland), followed by static incubation at 37 °C for 48 h. OD_600_ measurements were recorded every 4 h over the incubation period. To evaluate the growth inhibitory effects of FA, sub-inhibitory concentrations (¼ MIC and ½ MIC) were tested in parallel, with untreated cultures serving as controls. All experiments were conducted in triplicate [22].

## Antibiofilm activities of ferulic acid at sub-inhibitory concentrations

### Preventive antibiofilm assay

To assess the prophylactic antibiofilm activity of FA, 100 µL of each strong and moderate XDR *A. baumannii* (XDRAb) isolate (adjusted to OD_560_ = 0.02, approximately 1.0 × 10⁶ CFU/mL) in CAMHB supplemented with 1% glucose was inoculated into 96-well plates and incubated statically at 37 °C for 4 h. Then the plates were removed from the incubator and 100 µL of FA solution in media was added in a sequence in each well to give a final concentration equivalent to ¼ MIC & ½ MIC. Subsequently, plates were subjected to static incubation at 37 °C for an additional 24 h [23, 24]. Biofilm biomass was quantified using the crystal violet staining technique [20]. All experiments were conducted in triplicate. Wells without dissolved FA served as positive controls for biofilm formation. Biofilm biomass was quantified by measuring the OD at 595 nm. The Biofilm inhibition (%) was determined using the formula [25].

$$\%\:Biofilm\:inhibition=100-(\frac{OD\:sample}{OD\:positive\:control})\times100$$

### Microbial adhesion to hydrocarbons assay

Cell surface hydrophobicity (CSH), a key factor in microbial adhesion, was assessed for each strong biofilm-forming XDRAb isolate treated with and without FA using the microbial adhesion to hydrocarbons (MATH) assay. This method evaluates bacterial affinity for the hydrophobic hydrocarbon toluene, following the previously described protocol [26, 27]. Concisely, 1 mL of the test bacterial culture was placed into glass tubes with 250 *µ*L of toluene. The mixtures were vigorously vortexed for 2 min and allowed to stand at room temperature for 10 min to permit phase separation. The OD of the aqueous phase was then measured at 530 nm. The hydrophobicity index (%) was calculated using the following formula:

$$\%\:Hydrophobicity\:index=100-(\frac{Final\:OD\:after\:vortex\:}{Initial\:OD\:before\:vortex})\times\:100$$

### Therapeutic antibiofilm assay

To examine FA’s efficacy against established biofilms, strong and moderate XDRAb isolates were grown statically in 96-well flat base plates for 48 h to allow mature biofilm formation [20]. After discarding planktonic cells, 200 µL of FA solution in CAMHB medium at sub-inhibitory concentrations of ¼ MIC and ½ MIC were sequentially added to each well and cultured for an additional 24 h. Each experiment was conducted in triplicate, with wells containing CAMHB and the solvent 0.5% DMSO, devoid of dissolved FA, serving as the positive controls for biofilm formation. The protocol mentioned above was followed to quantify the antibiofilm potential of the tested FA solution [25].

### The effect of ferulic acid on the metabolic activity of biofilm bacteria

The MTT (3-(4,5-dimethylthiazol-2-yl)-2,5-diphenyltetrazolium bromide) assay was used to assess the metabolic activity of biofilm-embedded bacteria treated with FA [28]. As previously stated, preformed biofilm samples subjected to ¼ MIC & ½ MIC of FA treatment were gently rinsed twice and incubated with 100 µL of 0.05% MTT solution at 37 °C for 3 h in the dark. The MTT solution was then removed, and 100 µL of DMSO was added to dissolve the purple formazan crystals formed within metabolically active bacterial cells. Plates were gently agitated for 10 min to ensure complete solubilization, and absorbance was measured at 520 nm using a microplate reader. The optical density at 520 nm (OD₅₂₀) was used as an indicator of metabolic activity, where higher values represent greater metabolic activity. The percentage reduction in metabolic activity relative to the untreated control was calculated as follows:

$$\%\;Metabolic\;activity\;reduction=100\;-(\frac{OD_{520}\;treated}{OD_{520}\;positive\;control})\times\;100$$

### Quantification of exopolysaccharides

The influence of FA on EPS production was evaluated using strong biofilm-forming XDRAb isolates by applying a previously established methodology [29]. In summary, the 2-day-old preformed biofilm of strong biofilm-forming XDRAb isolates in 15-mL falcon tubes were incubated in Luria-Bertani (LB) broth (Oxoid, England) with and without FA at concentrations of ¼ MIC & ½ MIC. Following a 24-hour incubation at 37 °C, the cultures were centrifuged at 10,000 rpm for 15 min. The resulting pellets were resuspended in PBS and subjected to a second centrifugation under the same conditions for 30 min. The resulting supernatants were then mixed with an equal volume of ethyl alcohol and centrifuged again at 10,000 rpm for 30 min. To assess EPS production, 1 mL of the EPS-containing solution was carefully combined with 1 mL of cold 5% phenol and 5 mL of concentrated sulfuric acid, inducing the formation of a red color. To assess EPS quantity, the intensity of the color was measured spectrophotometrically at 490 nm. The percentage reduction of EPS following FA treatment was calculated in accordance with [30] :

$$\%\;EPS\;reduction=(\frac{OD\;control-OD\;treated}{OD\;control})\times\;100$$

### Microscopical assessment of antibiofilm activities of FA

A representative strong biofilm-forming XDRAb isolate (Ac88, exhibited the strongest biofilm-forming phenotype among all tested isolates, as indicated by the highest OD₅₉₅ values across repeated assays) was selected to visualize the impact of FA on established biofilms using light microscopy (LM), scanning electron microscopy (SEM), and confocal laser scanning microscopy (CLSM).

### Light microscopy

The biofilm eradication potential of FA was further confirmed using LM, as illustrated previously [31, 32]. In summary, 1 mL of bacterial suspension (10⁸ CFU/mL in tryptic soy broth supplemented with 1% glucose) was added to a 6-well microtiter plate containing 1 × 1 cm sterile glass coverslips. Post 48 h of static incubation at 37 °C to allow biofilm formation, planktonic cells were removed, and the wells were gently rinsed three times with normal saline. Then, 500 µL of FA (¼ MIC & ½ MIC) was added. A control was established using 500 µL of 0.5% DMSO alone. The plate was incubated statically at 37 °C for an additional 24 h. Following treatment, the coverslips were gently washed with PBS, stained with 0.1% crystal violet, and rinsed with deionized water to remove excess stain. After air drying, the coverslips were examined under a light microscope (LABOMED, CXL, USA) at 400× magnification.

### Scanning electron microscope analysis

The antibiofilm efficacy of FA against the established XDRAb biofilm was further evaluated using SEM, following a previously described protocol [22]. In short, the biofilms were produced on 1 × 1 cm size coverslips with all techniques, as stated above. After incubation, the biofilms were fixed with 2.5% glutaraldehyde at 37 °C for 30 min, followed by three washes with PBS solution. The samples were then dehydrated through a graded ethanol series, air-dried, and sputter-coated with gold. Morphological analysis of the biofilms was performed using a scanning electron microscope (S-34002 N SEM, Hitachi^®^, Tokyo, Japan).

### Confocal Laser Scanning Microscopy (CLSM)

The antibiofilm efficacy of FA against the established XDRAb biofilm was observed using a CLSM, as previously detailed [33]. The 8-well chamber slide (ibidi, Martinsried, Germany), utilized for biofilm development, was rinsed thrice with PBS to eliminate planktonic cells. The biofilms were then stained with 5 µL of acridine orange (AO) to visualize live cells (green fluorescence) and 5 µL of propidium iodide (PI) to detect dead cells (red fluorescence), followed by incubation in the dark for 15 min. The stained biofilms were visualized and analyzed using CLSM (DMi8; Leica Microsystem).

### Gene expression analysis using quantitative, real-time PCR

The impact of FA at ½ MIC on the expression of biofilm-associated genes (*abaI*, *bfmR*, *bap*, *csuE*, and *pgaB*) was assessed in four strong biofilm-producing XDRAb isolates. For this purpose, isolates were cultured in TSB supplemented with or without FA (½ MIC) in 15 mL Falcon tubes and incubated statically at 37 °C for 24 h. Bacterial cells were harvested by centrifugation at 3000 rpm for 5 min, and total RNA was extracted from the resulting biofilm pellets using the PureLink™ RNA Mini Kit (Thermo Scientific, USA), following the manufacturer’s protocol. RNA concentration and purity were measured by NanoDrop spectrophotometer (ND-1000). Complementary DNA (cDNA) was synthesized from total RNA using the First Strand cDNA Synthesis Kit (Thermo Scientific, USA). Quantitative real-time PCR (RT-qPCR) was performed using Power SYBR™ Green Master Mix (Thermo Scientific, USA) and gene-specific primers as previously described [7, 34, 35]. In both FA-treated and untreated samples, *16 S rRNA* was used as a housekeeping gene [36]. Primer sequences are demonstrated in Table 1. Data analysis was performed using Opticon Monitor software (version 3.0; Bio-Rad). Melting curve analysis was conducted for each gene to ensure the specificity and identity of the amplified products. The relative expression levels of the target genes were quantified using the 2^–ΔΔCT^ method, as described by [37], which is widely used for relative quantification in RT-qPCR analyses. The threshold cycle (CT) represents the cycle at which fluorescence exceeds a predetermined threshold, indicating detectable levels of amplified product.

Gene expression was normalized to the housekeeping gene *16 S rRNA*. The calculations were performed using the following equations:

- $$\begin{aligned}\Delta\mathrm{CT}\;\mathrm{(treated)}&\;=\;\mathrm{CT}\;\text{(target gene, treated)}\\&\;-\;\mathrm{CT}\;\text{(reference gene, treated)}\end{aligned}$$  
- $$\begin{aligned}\Delta\mathrm{CT}\;\mathrm{(control)}&\;=\;\mathrm{CT}\;\text{(target gene, control)}\\&\;-\;\mathrm{CT}\;\text{(reference gene, control)}\end{aligned}$$  
- $$\Delta\Delta\mathrm{CT}\;=\;\Delta\mathrm{CT}\;\mathrm{(treated)}\;-\;\Delta\mathrm{CT}\;\mathrm{(control)}$$  
- $$\mathrm{Relative}\;\mathrm{fold}\;\mathrm{change}\;=\;2^{-\Delta\Delta\mathrm{CT}}$$  

**Table 1:** Primer sequences used for evaluating gene expression in RT-qPCR analysis

Gene	Role [34, 38]	Sequence	Reference
*abaI*	Involved in the synthesis of N-acyl homoserine lactone, a key quorum-sensing molecule.	F 5′-CCC GCA GCA CGT AAT AAA CG-3′ R 5′-AGC AGT CAG GCT GTG TCA TC-3′	[34]
*bfmR*	Functions as a response regulator in the *bfmRS* two-component system and plays a master role in biofilm initiation.	F 5′-ATT CGT GCT TTG TTA CGC CG-3′ R 5′-GCG ATA AAA TAC GGC CAG CG-3′	[34]
*bap*	Encodes a biofilm-associated protein that facilitates intercellular adhesion and biofilm structural stability.	F 5′-TGC TGA CAG TGA CGT AGA ACC ACA-3′ R 5′-TGC AAC TAG TGG AAT AGC AGC CCA-3′	[7]
*csuE*	Part of the chaperone-usher pilus assembly system; mediates initial surface attachment and promotes biofilm maturation.	F 5′-CAT CTT CTA TTT CGG TCC C-3′ R 5′-CGG TCT GAG CAT TGG TAA-3′	[7]
*pgaB*	Encodes a polysaccharide N-deacetylase that modifies poly-β-(1→6)-N-acetylglucosamine (PNAG), contributing to biofilm matrix integrity.	F 5′-AAG AAA ATG CCT GTG CCG ACC A-3′ R 5′-GCG AGA CCT GCA AAG GGC TGA T-3′	[35]
*16 S rRNA*	Serves as an internal reference (housekeeping) gene for normalization of gene expression data.	F 5′-TGG CTC AGA TTG AAC GCT GGC GGC-3′ R 5′-TAC CTT GTT ACG ACT TCA CCC CA-3′	[36]

### In vitro cytotoxicity (MTT) assay

The cytotoxic potential of FA on the human skin fibroblast (HSF) normal cell line (National Research Centre, Cairo, Egypt) was assessed using the MTT assay, as previously described [39]. HSF cells were cultured in Dulbecco’s Modified Eagle Medium supplemented with 10% foetal bovine serum, 100 U/mL penicillin, and 100 µg/mL streptomycin, and maintained at 37 °C in a humidified atmosphere containing 5% CO₂. Serial two-fold dilutions of FA were prepared to test a range of concentrations. Confluent cell monolayers were seeded in 96-well microtiter plates and incubated for 24 h. The cells were then treated with the different concentrations of FA in triplicate and incubated for 48 h under standard conditions. After treatment, 20 µL of MTT solution (5 mg/mL) was added to each well and incubated for 4 h at 37 °C. Following incubation, the medium was gently aspirated, and 150 µL of DMSO was added to dissolve the formazan crystals. Plates were covered with aluminum foil and placed on an orbital shaker for 15 min. The absorbance was measured at 570 nm using a microplate reader (B.M.G. Labtech, FLUOstar Omega, Ortenberg, Germany). The half-maximal inhibitory concentration (IC₅₀), indicating the concentration of FA required to inhibit 50% of cell viability, was typically obtained by plotting % viability versus log_10_ concentration of FA using a non-linear regression curve fit. For subsequent assays, concentrations below IC₅₀ were selected as the safe range.

## In vivo studies

### Challenging microorganism

Clinical isolate *A. baumannii* Ac 82 was chosen for the in vivo evaluation test. It is a strong biofilm former, isolated from a wound clinical sample, and has a MAR index of 0.85, thereby reflecting a clinically relevant wound-associated XDRAb strain.

### Laborarory animals

A total of twenty male Wistar rats, aged six to eight weeks (body weight range: 140–165 g), were obtained from the animal facility at the Faculty of Veterinary Medicine, Cairo University (Cairo, Egypt). The animals were housed in temperature-controlled cages under standard laboratory conditions and a 12-h light/dark cycle, with *ad libitum* access to food and water. Prior to experimental procedures, a 7-day acclimatization period was implemented to ensure animal welfare. All housing, handling, and experimental protocols were conducted in strict accordance with institutional guidelines for animal care and use, following internationally recognized ethical standards. To minimize microbial contamination and ensure aseptic conditions, the cages were routinely disinfected with a 10% povidone-iodine solution, while bedding materials were replaced daily.

### Establishment of *A. baumannii* infection animal models

First, we made animals transiently neutropenic using intraperitoneal (I.P) injection of cyclophosphamide (150 mg/kg of body weight). Approximately 0.2 mL of the medication was injected on days 4 and 1 prior to the inoculation with the challenge strain Ac 82 (day 0) [40, 41]. Concisely, each rat was anesthetized by I.P injection of thiopental at a dose of 40 mg/kg body weight. It is a commonly accepted and technically feasible approach in rodent wound infection models providing rapid and short-duration anesthesia, and was used with freshly prepared thiopental at the recommended dilution administered in the lower abdominal quadrant to minimize irritation, noting that reported thiopental-related irritation is mainly associated with extravascular or intradermal leakage rather than proper I.P use, with continuous monitoring of anesthetic depth and adverse effects under institutional ethical approval (TP / RE/5/25 p-002) [42, 43]. The hair of the rats’ dorsal skin was removed by a depilatory cream and cleaned with povidone-iodine (10%) and ethanol (70%). One excisional wound, 10 mm in diameter, was performed on the dorsal section of the rat using a biopsy punch. Briefly, 5 min post-wounding, aliquots of 50 µL containing 10^8^ CFU/mL Ac82 cells in a PBS suspension were pipetted into the wound and allowed to absorb for 3 min. A circular cutout (30 mm in diameter) of transparent dressing (TegadermRoll; 3 M Health Care, St. Paul, MN) was placed over the wound and secured with tissue adhesive [44]. The rats were housed in individual cages. As previously reported [45, 46], a 24-hour incubation period was maintained between bacterial inoculation and successful infection model establishment. For each experiment, animals were randomly allocated into two treatment groups (*n* = 10 per group):

- Group 1 (vehicle control; 0.5% DMSO control): Infected animals receiving topical application of vehicle control (0.5% v/v DMSO in sterile PBS) without therapeutic intervention to confirm FA-specific effects beyond solvent;
- Group 2 (treatment group): Infected animals treated topically with FA at the predetermined MIC dose (512 μg/mL).

An untreated infected group was not included as the study focus was on FA efficacy relative to the vehicle. All therapeutic interventions started 24 h post-inoculation, with single daily topical administrations maintained for six consecutive days. The positive control group received parallel vehicle treatments following an identical administration schedule. The size of the wound was monitored and measured to assess the impact of FA on wound closure, and the percentage of wound closure was determined according to the following formula:

$$\begin{aligned}&\text{Wound closure}\,{\%}\\&=\frac{\text{initial wound size}-\text{wound size at the time of measurement}}{\text{initial wound size}}\times100\end{aligned}$$

On day 7, six randomly selected rats from each experimental group were euthanized by decapitation under anesthesia using I.P administration of thiopental (40 mg/kg body weight). Full-thickness wound tissues, including a 2–5 mm peripheral margin of surrounding skin, were aseptically excised for concurrent histopathological analysis and quantitative bacteriological assessment. The bacterial load in each 1 g wound tissue sample was quantified by rinsing the tissue well with 1 mL PBS and performing serial ten-fold dilutions. Aliquots (100 µL) of each dilution were plated onto MHA plates, which were incubated at 37 °C for 24 h. Colonies were then enumerated, and bacterial counts were calculated as colony-forming units per milliliter (CFU/mL) according to the following formula [47]. This yields concentration in the rinsing solution (1mL) and divide by 1 (tissue gram weight) for CFU/g tissue.

$$\begin{aligned}&Bacterial\:count\:in\:the\:original\:rinsate/mL\\&=\frac{numbers\:of\:colonies\times\:dilution\:factor}{inoculated\:volume}\end{aligned}$$

All specimens were immediately fixed in 10% neutral buffered formalin for 24 h at 4 °C, then transported to the Pathology Department, College of Medicine, Tanta University for processing. Following fixation, tissues were dehydrated, embedded in paraffin, and sectioned at 3–4 μm thickness. Sections were subsequently stained with hematoxylin and eosin (H&E) for histological evaluation.

## Statistical analysis

All experiments were conducted in triplicate, with results expressed as mean ± standard deviation (SD). Between-group comparisons used unpaired *t*-tests, while multiple-group analyses employed one-way or two-way ANOVA with appropriate post hoc tests. Statistical analyses were performed using GraphPad Prism v9.0; *p* < 0.05 was considered statistically significant.

## Results

### Isolation of extensively drug-resistant *Acinetobacter baumannii* from cancer patients

**Fig. 1:**
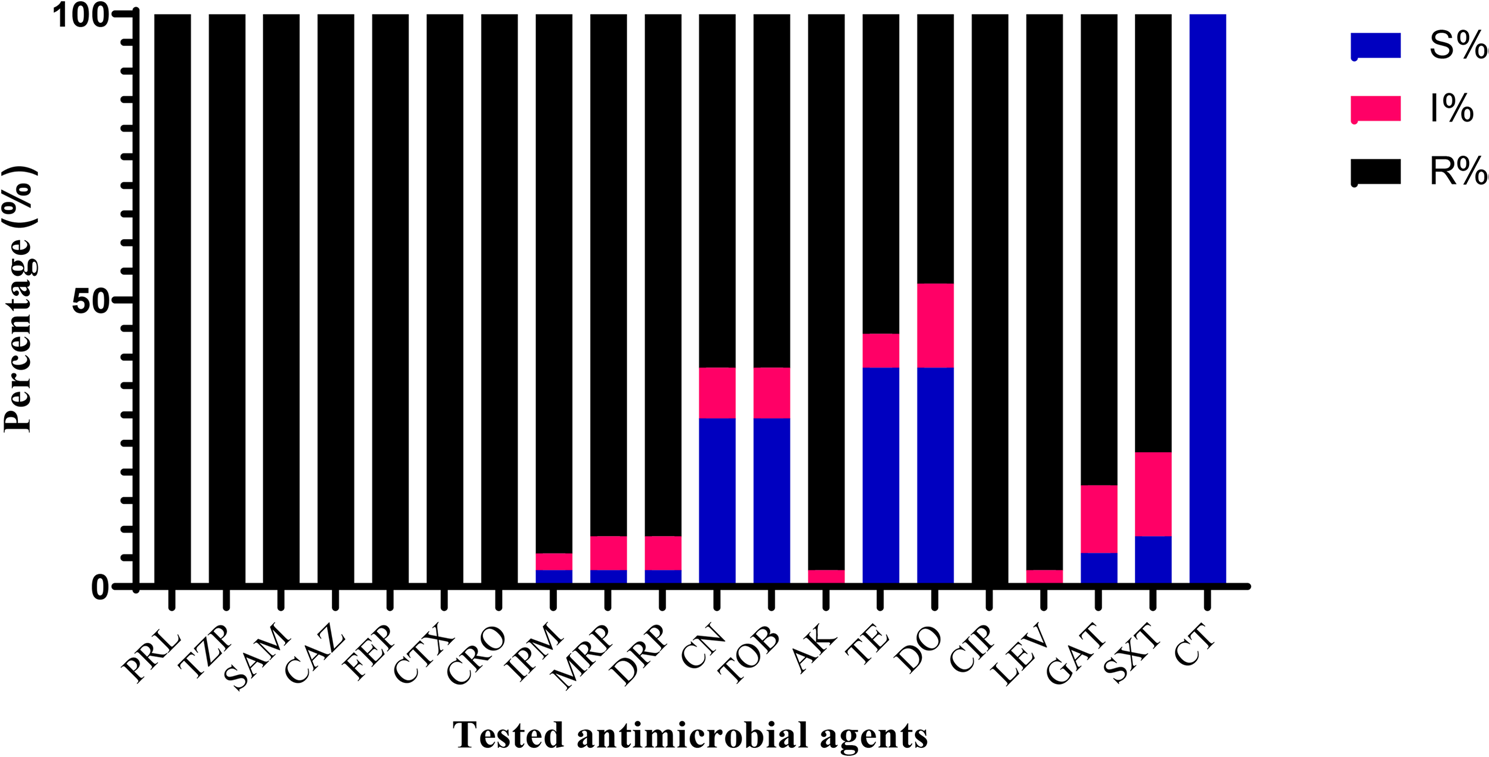
Incidence of resistance of tested *A. baumannii* to different antimicrobials. R, I, and S refer to resistant, intermediate, and sensitive to antibiotics, respectively. piperacillin (PRL, 100 µg), piperacillin-tazobactam (TZP, 100/10 µg), ampicillin-sulbactam (SAM, 10/10 µg), ceftazidime (CAZ, 30 µg), cefepime (FEP, 30 µg), cefotaxime (CTX, 30 µg), ceftriaxone (CRO, 30 µg), imipenem (IPM, 10 µg), meropenem (MRP, 10 µg), doripenem (DRP, 10 µg), gentamicin (CN, 10 µg), tobramycin (TOB, 10 µg), amikacin (AK, 30 µg), tetracycline (TE, 30 µg), doxycycline (DO, 30 µg), ciprofloxacin (CIP, 5 µg), levofloxacin (LEV, 5 µg), gatifloxacin (GAT, 5 µg), trimethoprim/sulfamethoxazole (SXT, 1.25/23.75 µg), colistin (CT)

The XDR isolates were 34% (*n* = 34). These isolates were recovered from different biological specimens. They showed the highest frequencies among endotracheal aspirates (*n* = 19, 55.88%), whereas strains collected from sputum (*n* = 2, 5.88%), blood (*n* = 7, 20.59%), and wound (*n* = 6, 17.65%) samples were relatively lower as shown in Table 2. Antibiotic susceptibility of the selected XDR *A. baumannii* **(**XDRAb**)** isolates (*n* = 34) was examined against a panel of twenty antibiotics belonging to nine categories, as shown in Table 2. All these isolates (100%) were shown to be resistant to piperacillin, piperacillin-tazobactam, ampicillin-sulbactam, ceftazidime, cefepime, cefotaxime, ceftriaxone, and ciprofloxacin. On the other hand, colistin was found to be active against all these isolates. At the same time, the percentages of antimicrobial resistance to the rest of the tested antimicrobial agents ranged from 47.1% to 97.1%, as shown in Fig. 1.

**Table 2:**
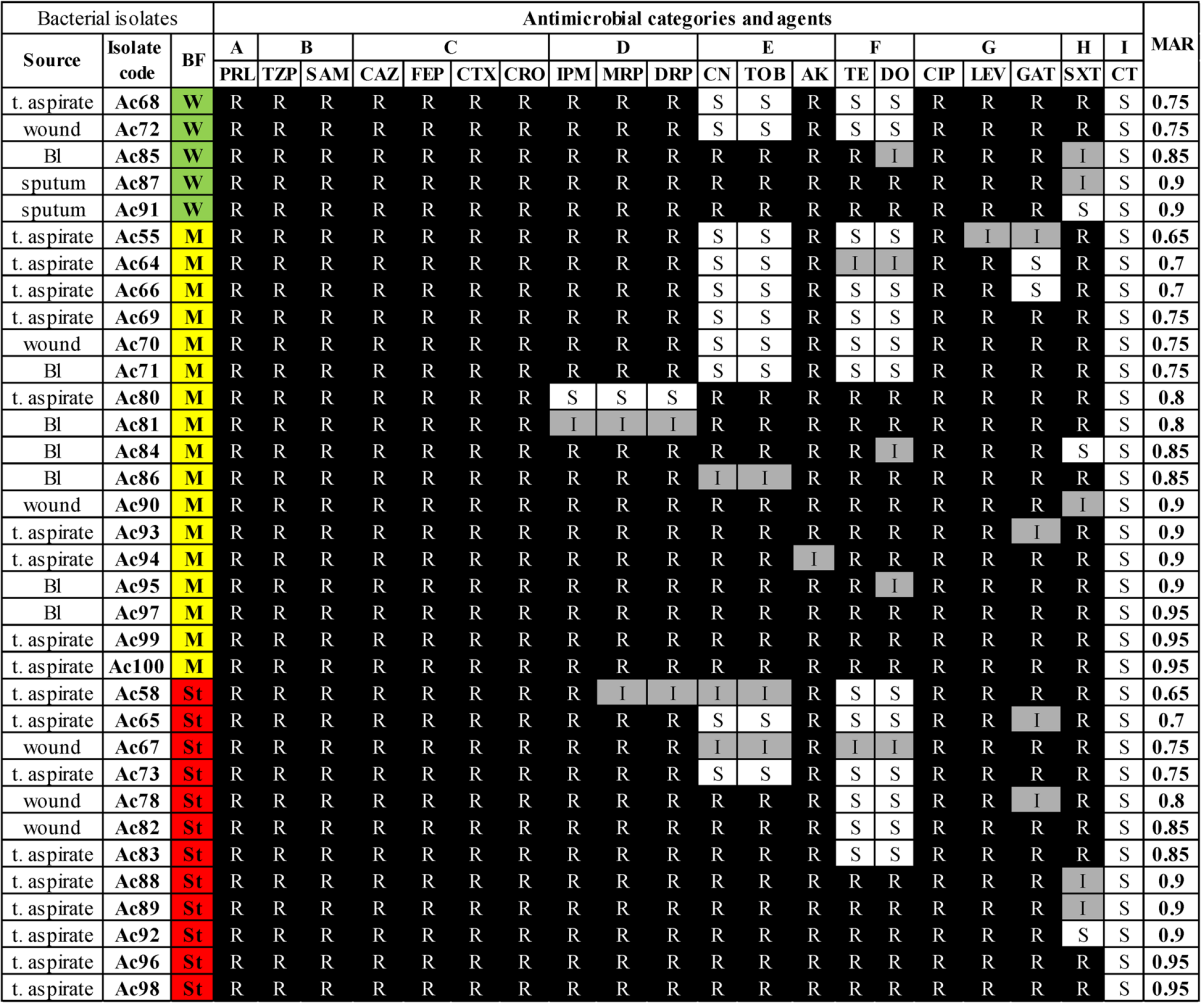
Antibiotic resistance profiles of 34 clinical XDR *Acinetobacter baumannii* isolates categorized by biofilm formation capacity

Biofilm formation capacity: (*W *weak, *M* moderate, *St *strong)

Susceptibility interpretations: *R *resistant, *I *intermediate, *S *sensitive

Antibiotics tested (disk content): PRL (piperacillin, 100 µg), TZP (piperacillin-tazobactam, 100/10 µg), SAM(ampicillin-sulbactam, 10/10 µg), CAZ (ceftazidime, 30 µg), FEP (cefepime, 30µg), CTX (cefotaxime, 30 µg), CRO (ceftriaxone, 30 µg), IPM (imipenem, 10 µg),MRP (meropenem, 10 μg), DRP (doripenem, 10 μg), CN (gentamicin, 10 μg), TOB(tobramycin, 10 µg), AK (amikacin, 30 µg), TE (tetracycline, 30 µg), DO(doxycycline, 30 µg), CIP (ciprofloxacin, 5 µg), LEV (levofloxacin, 5 µg), GAT(gatifloxacin, 5 µg), SXT (trimethoprim/sulfamethoxazole, 1.25/23.75 µg), CT(colistin)

Antibiotic categories (A-I) A: Penicillins, B: β-lactam combination agents, C: Cephems, D: Carbapenems, E: Aminoglycosides, F:Tetracyclines, G: Fluoroquinolones, H: Folate pathwayantagonists, I: Lipopeptides

**t. aspirates**: tracheal aspirates **MAR**: Multiple Antibiotic Resistance index

### Biofilm-forming capacity of XDR *A. baumannii* isolates

**Fig. 2:**
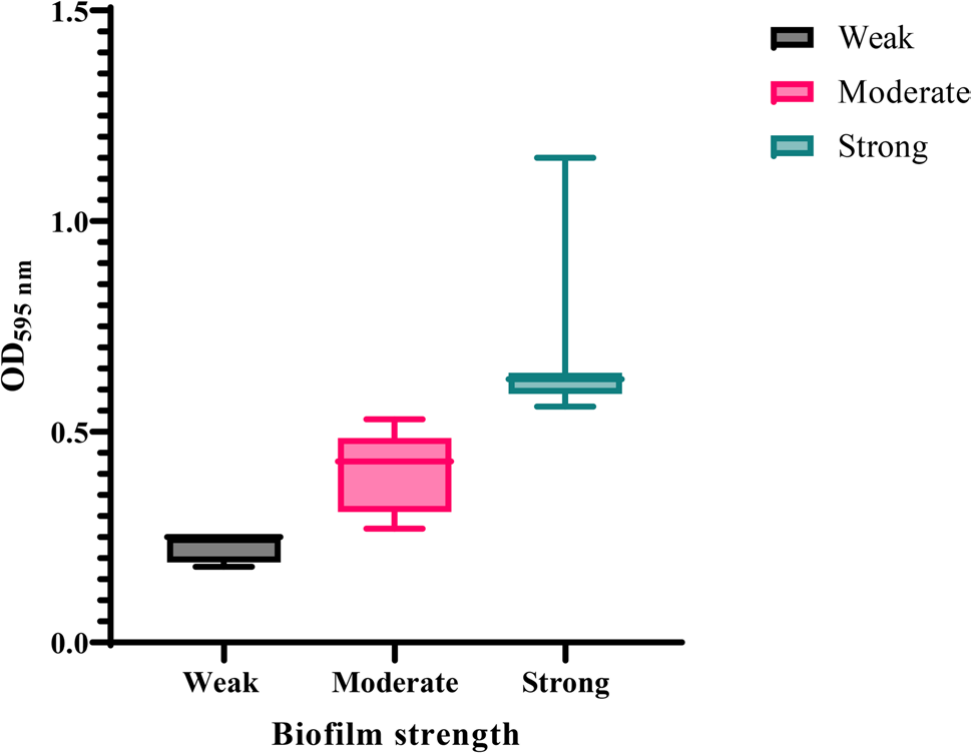
Biofilm formation capacity of the tested 34 XDR *Acinetobacter baumannii* isolates, categorized as weak, moderate, or strong producers based on optical density (OD_595_) measurements, showing the distribution pattern among tested isolates

The crystal violet microtiter plate assay classified the 34 XDRAb isolates into distinct biofilm-forming phenotypes (Table 2). Based on the measured OD_595_ values, the cutoff OD (ODc) was calculated as 0.13. Isolates were then classified according to their mean OD_595_ as follows: non-biofilm formers (OD_595_ ≤ 0.13), weak biofilm formers (0.13 < OD_595_ ≤ 0.26), moderate biofilm formers (0.26 < OD_595_ ≤ 0.52), and strong biofilm formers (0.52 < OD_595_), as depicted in Fig. 2. Accordingly, none of the isolates were non-biofilm formers, 5 isolates (14.7%) were weak, 17 isolates (50.0%) were moderate, and 12 isolates (35.3%) were strong biofilm formers. The reference strain *A. baumannii* ATCC 19,606 had an OD_595_ of 0.85, consistent with a strong biofilm-forming phenotype under the same assay conditions.

### Antibacterial activities of ferulic acid

Ferulic acid (FA) inhibited growth of XDRAb isolates with MICs ranging from 512 to 1024 µg/mL. The MIC was 512 µg/mL for 31 isolates and 1024 µg/mL for 3 isolates.

### Impact of sub-inhibitory concentrations of ferulic acid on bacterial growth rate

**Fig. 3:**
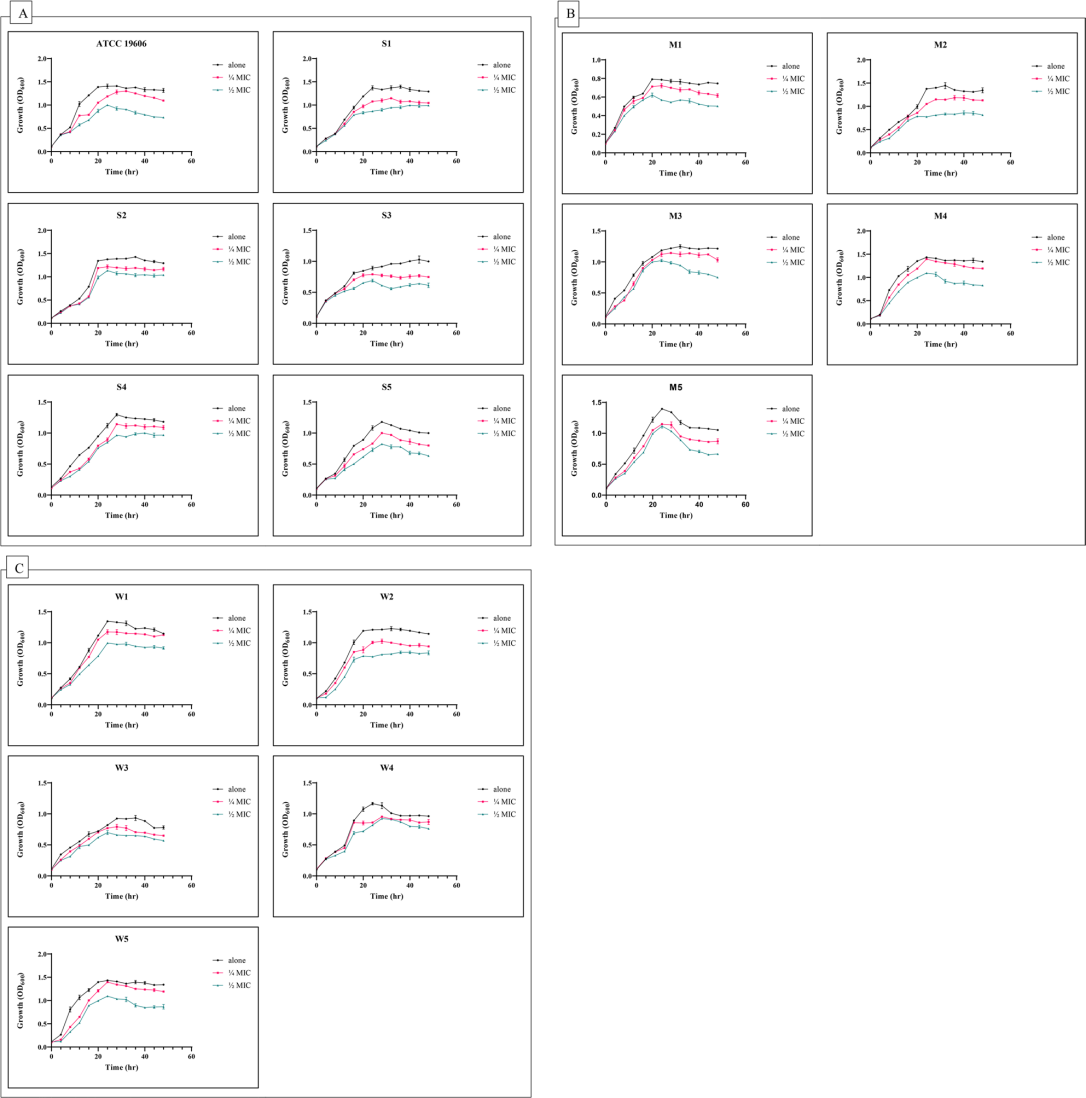
**A-C** Bacterial growth curves of selected *Acinetobacter baumannii* isolates in the presence of sub-inhibitory concentrations (¼ MICs and ½ MICs) of ferulic acid, (S) Strong biofilm formers, (M) Moderate biofilm formers, (W) Weak biofilm formers, each compared with their corresponding untreated controls. Bacterial growth was monitored by measuring optical density at 600 nm (OD₆₀₀) over 48 h. Data represent mean values from three independent experiments; error bars represent SD. No statistically significant differences were observed between treated and untreated groups (*p* > 0.05, one-way ANOVA with Dunnett’s post hoc test)

In this experiment, 5 isolates from each of strong, moderate, and weak biofilm-forming XDRAb isolates were selected for studying the effect of subMIC (¼ MIC and ½ MIC) of FA on the growth rate of the tested isolates. Simultaneously, ATCC 19606 reference strain was tested as a control. As shown in Fig.3, only minor growth pattern alterations (*p* > 0.05) were observed between the control and FA-treated isolates, indicating that sub-MIC of FA generally does not affect the planktonic growth of the tested isolates during biofilm development. Statistical analysis was performed using one-way ANOVA followed by Dunnett’s post hoc test, with *p* > 0.05 considered non-significant. 

## Antibiofilm activities of ferulic acid at sub-inhibitory concentrations

### Preventive antibiofilm assay

**Fig. 4:**
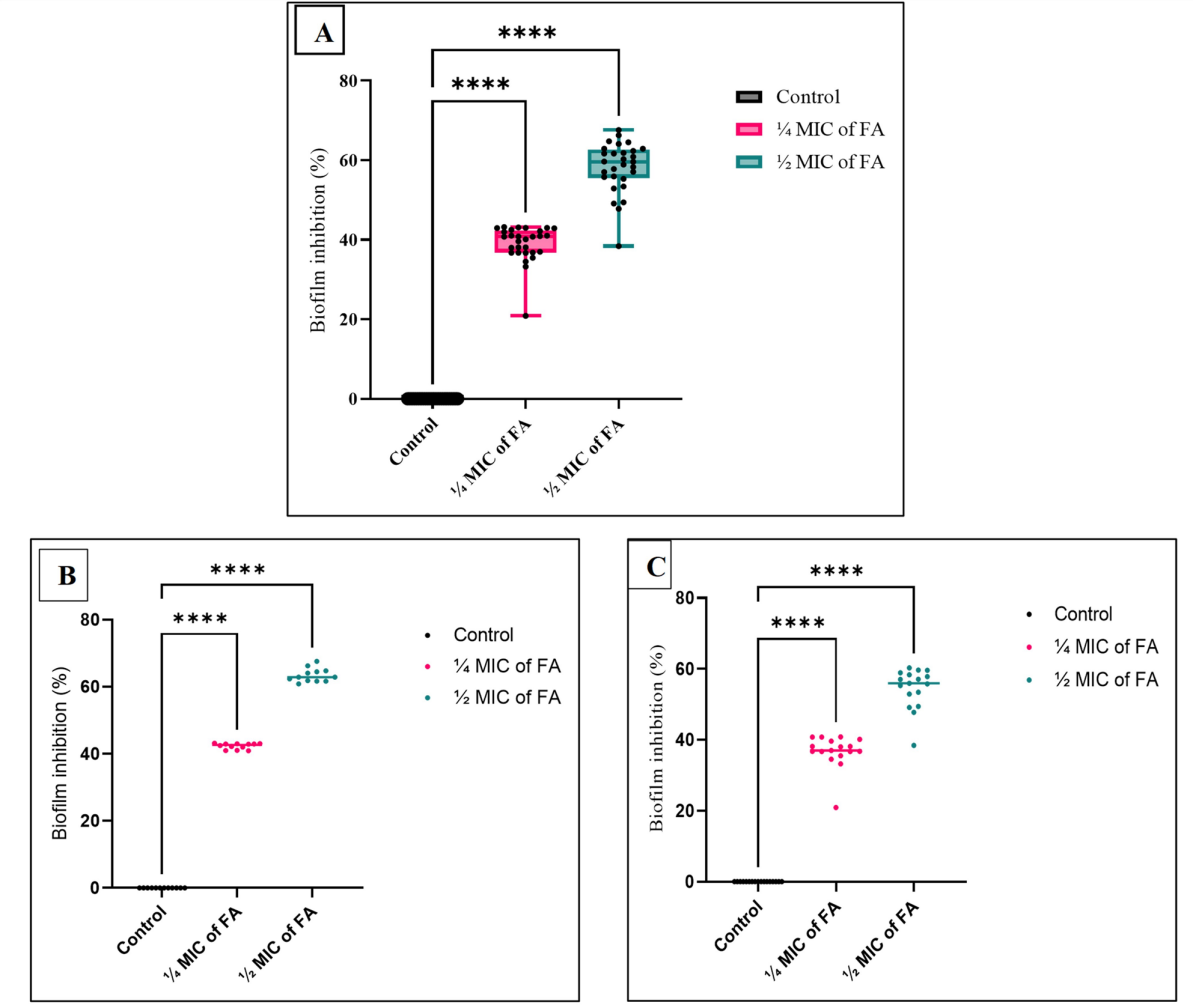
Preventive antibiofilm activity of sub-inhibitory ferulic acid (FA) concentrations (¼ MIC and ½ MIC) against XDR *Acinetobacter baumannii* clinical isolates. **A** Distribution of biofilm inhibition percentages among tested XDRAb isolates treated with ¼ MIC and ½ MIC of FA compared with untreated controls, presented as box-and-whisker plots with individual data points (black dots) overlaid. Boxes represent the interquartile range, center lines indicate medians, and whiskers denote minimum and maximum values. **B–C** Biofilm inhibition profiles of isolates stratified according to biofilm-forming capacity: strong (**B**, *n* = 12) and moderate (**C**, *n* = 17) biofilm formers. Scatter plots show individual isolate responses, with horizontal lines indicating mean inhibition values. Statistical analysis was performed using one-way ANOVA followed by Dunnett’s post hoc test. ****p < 0.0001 versus untreated control

Sub-inhibitory concentrations (¼ MIC and ½ MIC) of FA significantly inhibited biofilm formation in both strong and moderate biofilm-forming XDRAb isolates (*p* < 0.05; Fig. 4A). The inhibitory effect was dose-dependent, with ½ MIC producing the greatest reduction in biofilm biomass (67.6%), while ¼ MIC resulted in up to 43.2% inhibition relative to untreated controls. Statistical analysis using one-way ANOVA followed by Dunnett’s post hoc test confirmed that both concentrations differed significantly from the control group. Stratified analysis revealed that ¼ MIC and ½ MIC of FA significantly reduced biofilm formation in both strong and moderate biofilm formers (*p* < 0.05; Fig. 4B-C), with a trend toward greater inhibition among strong biofilm formers. Notably, treatment with these sub-MIC concentrations did not significantly affect planktonic growth rates (*p* > 0.05; Fig. 3), indicating that the observed antibiofilm activity was independent of growth inhibition.

### Microbial Adhesion to Hydrocarbons (MATH) assay

**Fig. 5:**
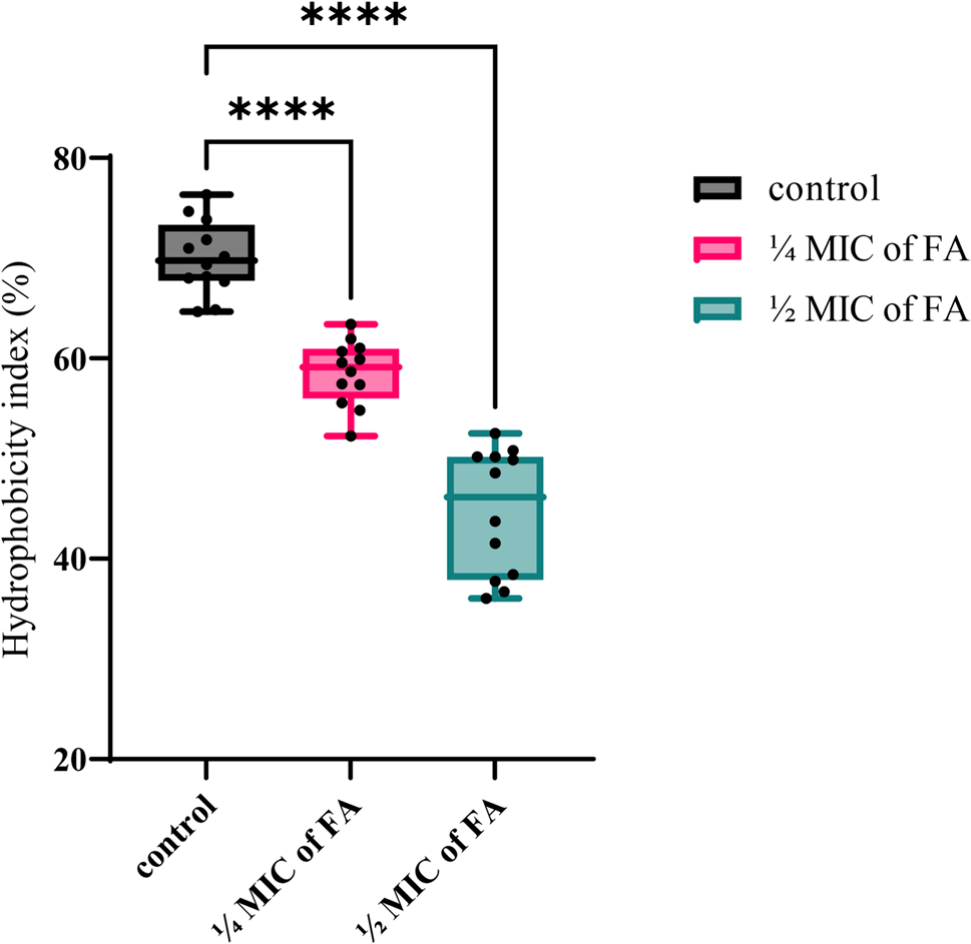
The impact of sub-inhibitory concentrations (¼ MIC and ½ MIC) of ferulic acid (FA) on cell surface hydrophobicity was evaluated in strong biofilm-forming XDRAb isolates (*n* = 12). Box-and-whisker plots display the distribution of hydrophobicity index percentages across the tested isolates treated with ¼ MIC or ½ MIC of FA compared to untreated controls. Box boundaries represent the 25th and 75th percentiles (interquartile range), horizontal lines within boxes indicate median values, and whiskers extend to minimum and maximum observations. Individual data points (black dots) are overlaid to illustrate the actual distribution of hydrophobicity index percentages among the tested isolates. Statistical analysis was performed using one-way ANOVA followed by Dunnett’s post hoc test; ****p < 0.0001 vs. untreated control

The MATH assay was employed to investigate whether the anti-biofilm activity of FA involves alterations in cell surface hydrophobicity. Results indicated a marked reduction in the hydrophobicity index of XDRAb isolates treated with ¼ MIC and ½ MIC of FA, reaching 63.4% and 52.5%, respectively, compared to 76.3% in untreated controls (Fig. 5). This progressive decline indicates that FA markedly reduces the affinity of XDRAb cells for the hydrophobic phase, suggesting impaired ability to initiate surface attachment and biofilm formation.

### Therapeutic antibiofilm assay

**Fig. 6:**
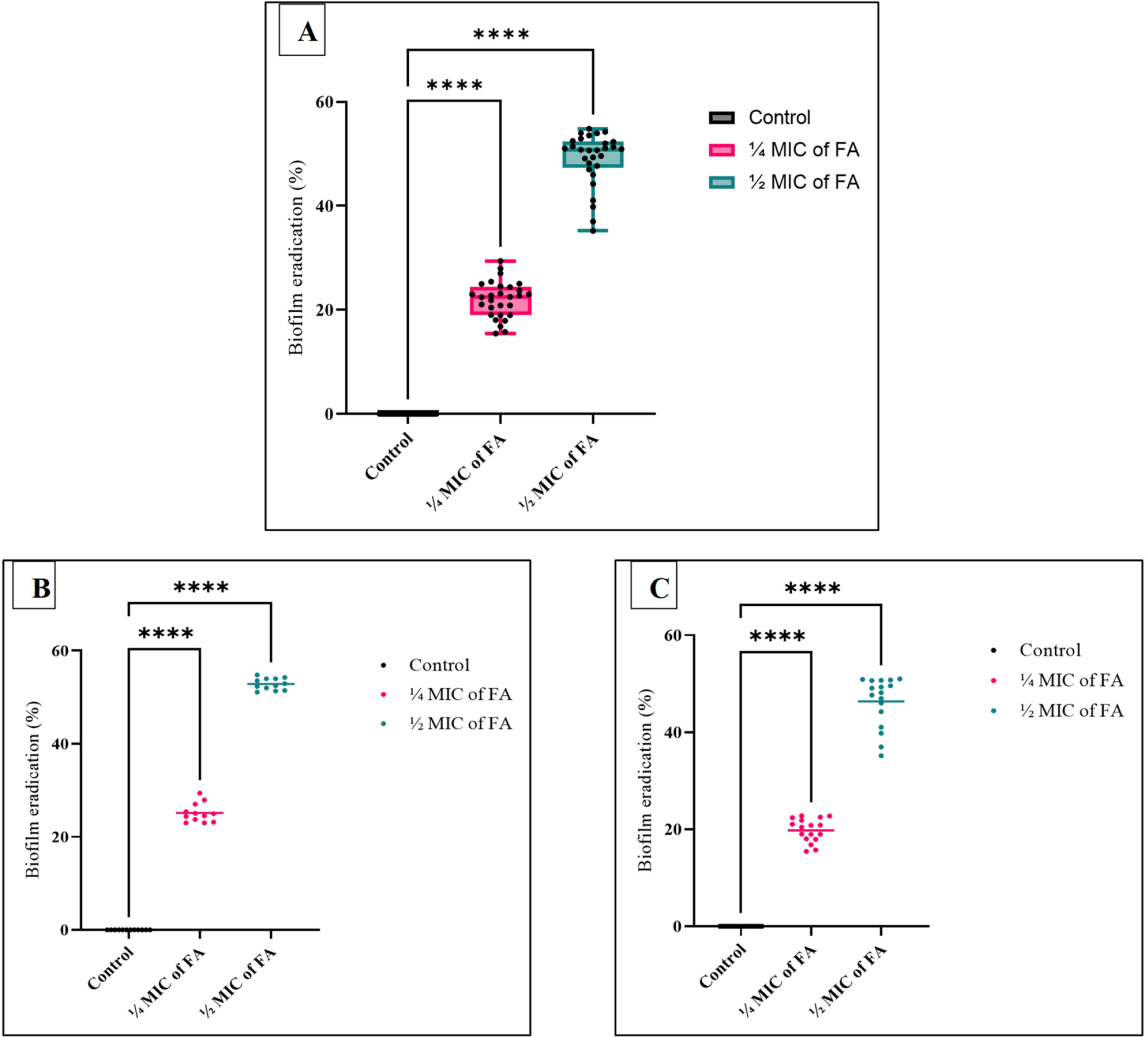
Therapeutic antibiofilm activity of sub-inhibitory concentrations of ferulic acid (FA) (¼ MIC and ½ MIC) against clinical XDR *Acinetobacter baumannii* isolates. **A** Distribution of biofilm inhibition percentages among tested XDRAb isolates treated with ¼ MIC and ½ MIC of FA compared with untreated controls, shown as box-and-whisker plots with individual data points overlaid (black dots). Boxes represent the interquartile range, center lines indicate medians, and whiskers denote minimum and maximum values. **B-C** Eradication of pre-established biofilms in isolates stratified by biofilm-forming capacity: strong biofilm formers (**B**, *n* = 12) and moderate biofilm formers (**C**, *n* = 17). Scatter plots display individual isolate responses, with horizontal lines indicating mean eradication values. Statistical analysis was performed using one-way ANOVA followed by Dunnett’s post hoc test. ****p < 0.0001 versus untreated control

Pre-established 48 h XDRAb biofilms were exposed to ¼ MIC & ½ MIC of FA using a microtiter plate assay. Tested biofilms were significantly disrupted following exposure to sub-inhibitory concentrations of FA (¼ MIC and ½ MIC) (*p* < 0.05; Fig. 6A). Treatment with ½ MIC produced the greatest eradication effect, achieving a maximum reduction of 54.8%, whereas ¼ MIC resulted in a 29.4% decrease in biofilm biomass compared with untreated controls. Stratification by biofilm-forming capacity revealed that both concentrations significantly reduced mature biofilms in both strong and moderate biofilm-forming isolates (*p* < 0.05; Fig. 6B-C). Across all biofilm phenotypes, eradication efficiency was consistently higher at ½ MIC than at ¼ MIC.

### The effect of ferulic acid on the metabolic activity of biofilm bacteria

**Fig. 7:**
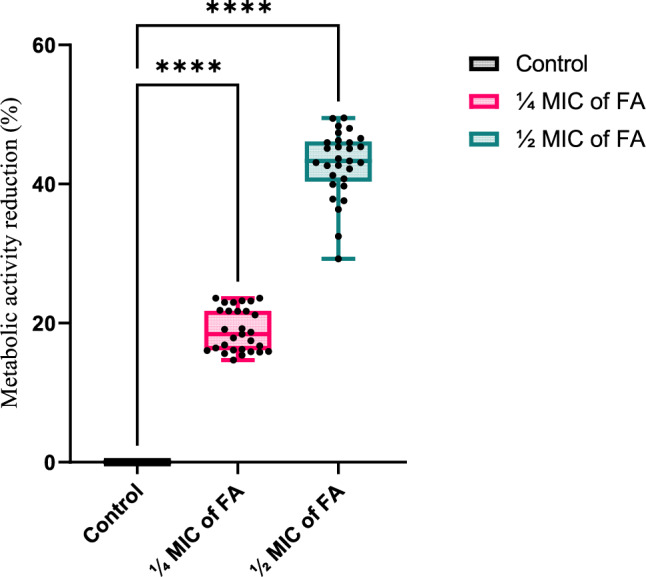
Box-and-whisker plots illustrate the distribution of metabolic activity reduction percentages among tested XDR *Acinetobacter baumannii* isolates treated with sub-inhibitory concentrations of ferulic acid (FA) (¼ MIC or ½ MIC) relative to untreated controls. Boxes represent the interquartile range (25th–75th percentiles), central lines denote median values, and whiskers indicate minimum and maximum observations. Individual data points (black dots) are overlaid to display isolate-level variability in metabolic activity reduction. Statistical significance was assessed using one-way ANOVA followed by Dunnett’s post hoc test. ****p < 0.0001 versus untreated control

The effect of ¼ MIC and ½ MIC FA on the metabolic activity of mature (2-day-old) biofilm-associated bacteria was evaluated using the thiazolyl blue tetrazolium bromide (MTT) assay (Fig. 7). Both concentrations significantly reduced bacterial metabolic activity (*p* < 0.05), with mean reductions of up to 23.6% and 48.3% for ¼ MIC and ½ MIC, respectively.

### Quantification of exopolysaccharides

**Fig. 8:**
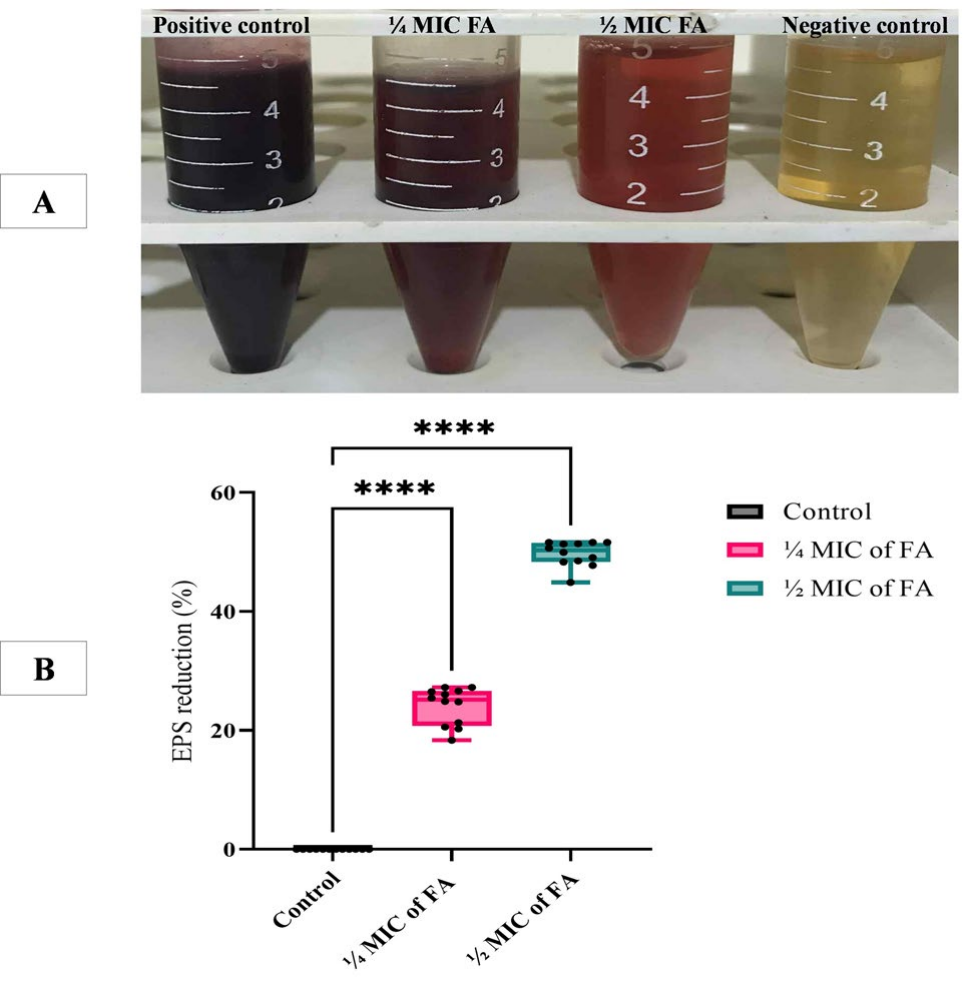
Effect of ¼ MIC and ½ MIC of ferulic acid (FA) on exopolysaccharides (EPS) production by strong biofilm-forming XDRAb isolates (*n* = 12), assessed via the phenol-sulfuric acid method. **A** Representative images showing differences in red color intensity. **B** Box-and-whisker plots illustrate the distribution of EPS reduction percentages among tested isolates treated with sub-inhibitory concentrations of FA (¼ MIC or ½ MIC) relative to untreated controls. Boxes represent the interquartile range (25th–75th percentiles), central lines denote median values, and whiskers indicate minimum and maximum observations. Individual data points (black dots) are overlaid to show isolate-level variability in EPS reduction. Statistical significance was assessed using one-way ANOVA followed by Dunnett’s post hoc multiple comparison test. ****p < 0.0001 versus untreated control

Using the phenol-sulfuric acid method to elucidate the mechanism by which FA could disrupt the established XDRAb biofilm, we evaluated its impact on exopolysaccharides (EPS) production, an essential component of the biofilm matrix. Treatment with ¼ MIC and ½ MIC of FA resulted in significant reductions in EPS content by 26.5% and 51.6%, respectively, as illustrated in Fig. 8.

### Microscopy analysis of the antibiofilm effects of ferulic acid

To visually assess the impact of FA on preformed XDRAb biofilms, light microscopy (LM), scanning electron microscopy (SEM), and confocal laser scanning microscopy (CLSM) were performed on a 48-hour-old biofilm of a representative strong biofilm-forming isolate (Ac88). LM analysis at 400× magnification revealed a notable reduction in biomass in treated samples. SEM imaging demonstrated dense cell layers in the untreated control, while the ¼ MIC-treated biofilm appeared rough with clumped cells, and ½ MIC-treated samples exhibited sparse, individually dispersed cells.

**Fig. 9:**
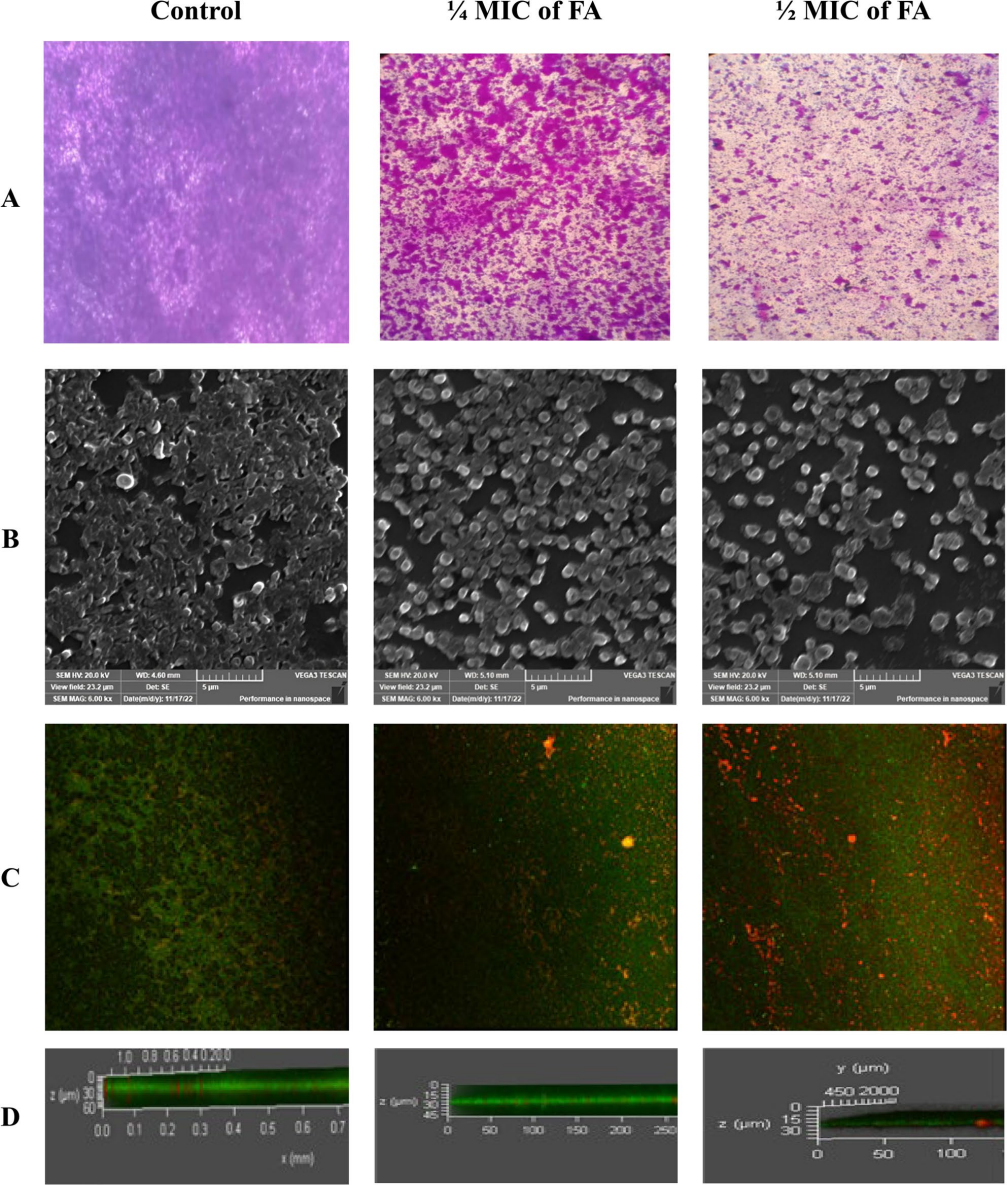
**A** Images of microscopic observations of the tested isolates using a bright field light microscope at a magnification of 400 ×. **B** SEM micrographs of biofilm structures (scale bar = 5 μm). **C–D** CLSM images display the viability and architecture of biofilms using AO/PI double staining in both 2D and 3D views, respectively

**Fig. 10:**
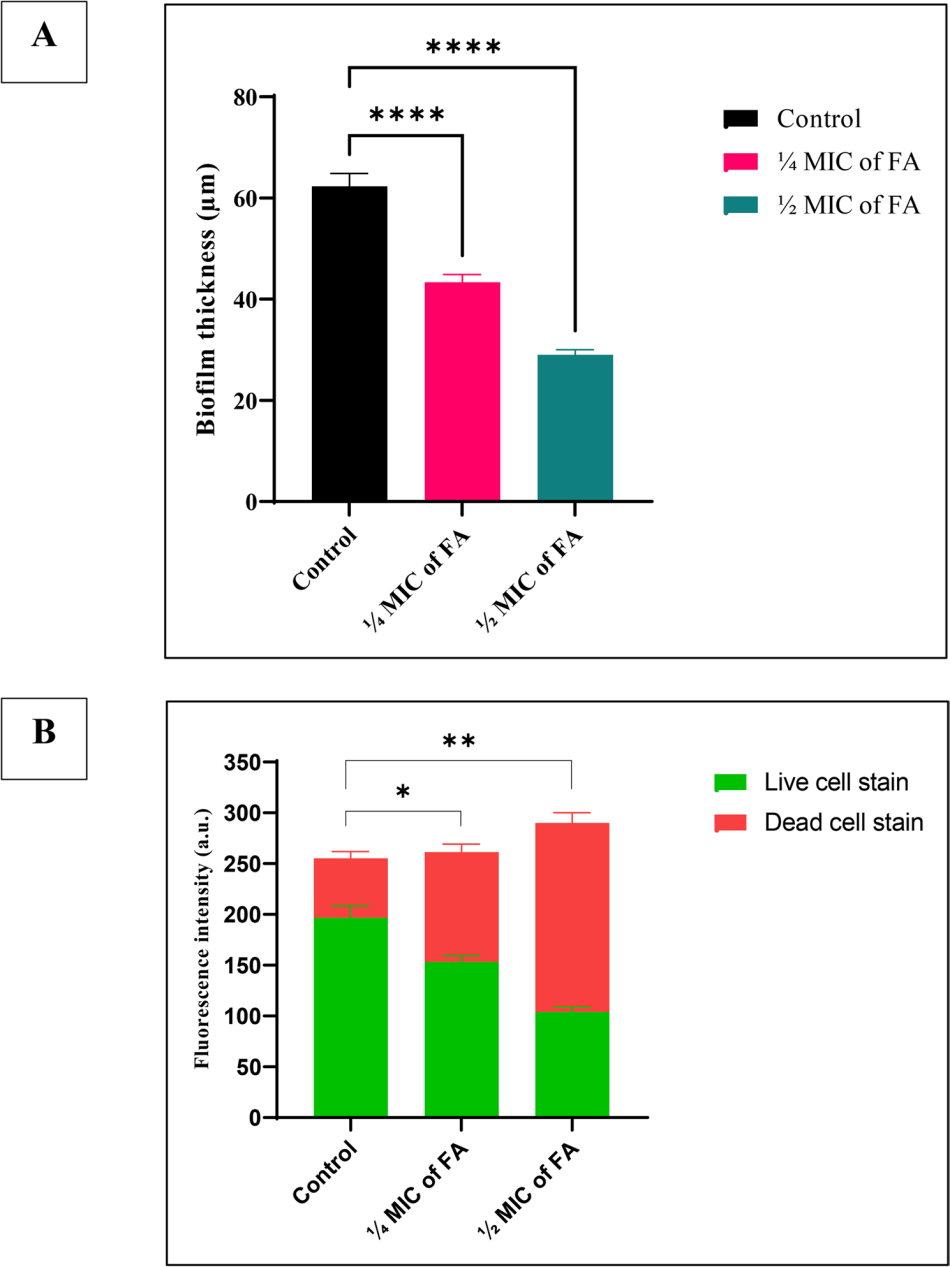
**A** Quantitative analysis of biofilm thickness derived from CLSM Z-stack measurements after treatment of a 48-h established biofilm of a representative strong biofilm former XDR *Acinetobacter baumannii* isolate (Ac88) with sub-inhibitory concentrations of ferulic acid (FA). Data are presented as mean ± SD. Statistical significance was assessed using one-way ANOVA followed by Dunnett’s post hoc test. ****p < 0.0001 versus untreated control. **B** Quantitative analysis of fluorescence intensity of biofilm-embedded cells following fluorescent staining. Fluorescence intensity is expressed as mean ± SD in arbitrary units (a.u). Statistical analysis was performed using two-way ANOVA followed by Dunnett’s post hoc multiple comparison test versus the untreated control. *p < 0.05; **p < 0.01

CLSM imaging using acridine orange (AO) and propidium iodide (PI) staining confirmed a decline in both viability and biofilm thickness post-treatment. Specifically, biofilm thickness was reduced by approximately 25% and 50% following treatment with ¼ MIC and ½ MIC of FA, respectively (Figs. 9 and 10).

### Effect of ferulic acid (½ MIC) on expression of biofilm-related genes

**Fig. 11:**
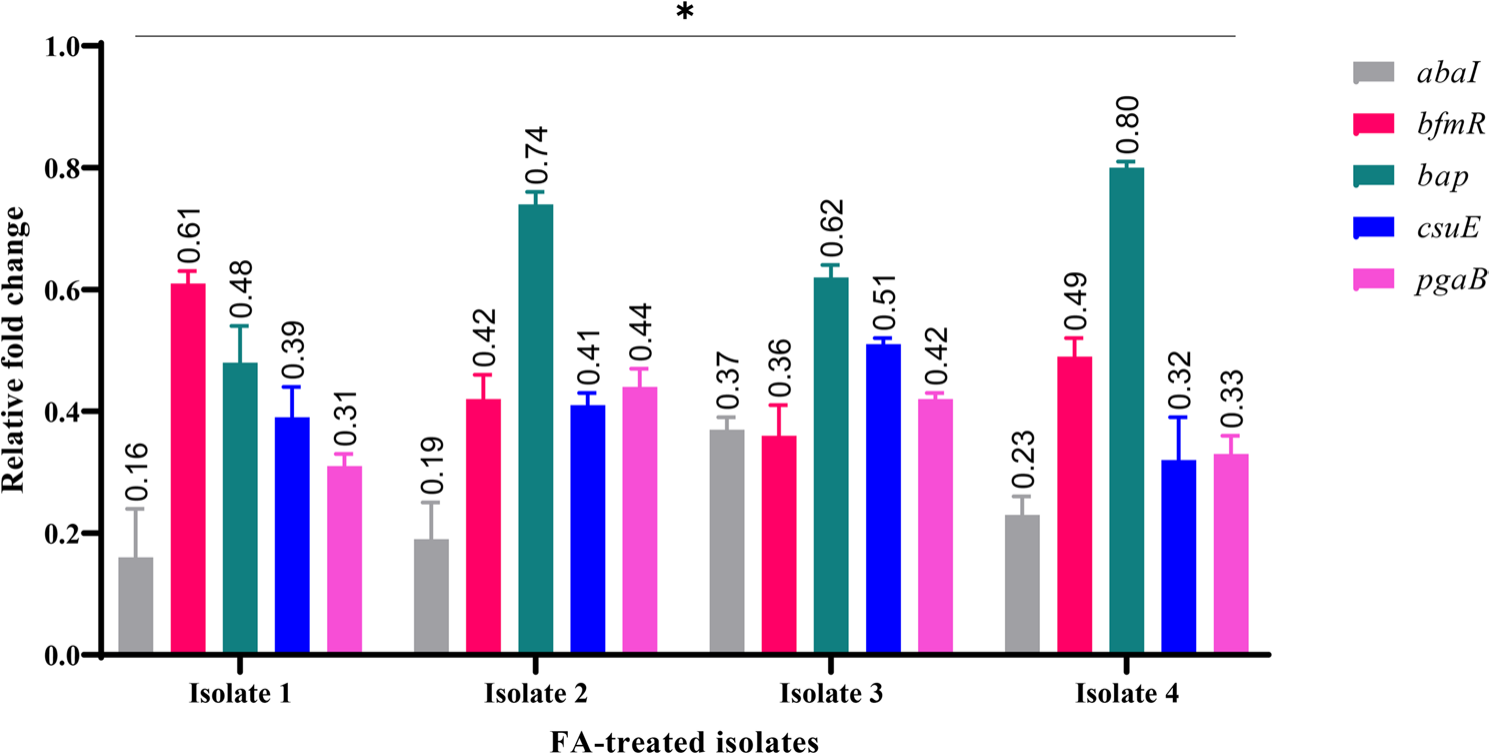
Relative fold changes in biofilm-associated gene expression following ferulic acid (FA) treatment at ½ MIC in strong biofilm-forming XDR *Acinetobacter baumannii* isolates (*n* = 4). Data represent mean fold change ± SD for *abaI*, *bfmR*, *bap*, *csuE*, and *pgaB* (2^–ΔΔCT^, normalized to *16 S rRNA* and untreated controls set to 1). Asterisk (*) indicates *p* < 0.05 vs. respective untreated control (*t*-test per isolate/gene)

Quantitative real-time PCR (RT-qPCR) was conducted to evaluate the transcriptional alterations in biofilm-associated genes (*abaI*,* bfmR*,* bap*,* csuE*, and *pgaB*) following exposure to FA at ½ MIC, compared to untreated controls. Exposure to FA at ½ MIC elicited significant downregulation (*p* < 0.05) of all tested biofilm-associated genes across four strong biofilm-forming XDRAb isolates, as shown in Fig. 11. Quorum sensing regulator *abaI* showed the most pronounced repression, with expression reduced to 0.16 ± 0.08, 0.19 ± 0.06, 0.37 ± 0.02, and 0.23 ± 0.03-fold of untreated controls (63–84% decrease). Pili-associated *csuE* (0.32 ± 0.07 to 0.51 ± 0.01-fold; 49–68% decrease) and exopolysaccharide synthesis gene *pgaB* (0.31 ± 0.02 to 0.44 ± 0.03-fold; 56–69% decrease) exhibited strong suppression, while regulator *bfmR* (0.36 ± 0.05 to 0.61 ± 0.02-fold; 39–64% decrease) and adhesin *bap* (0.48 ± 0.06 to 0.80 ± 0.01-fold; 20–52% decrease) displayed moderate downregulation. All changes were statistically significant (*t*-test, *p* < 0.05 vs. untreated controls per isolate/gene).

### In vitro cytotoxicity (MTT) assay

**Fig. 12:**
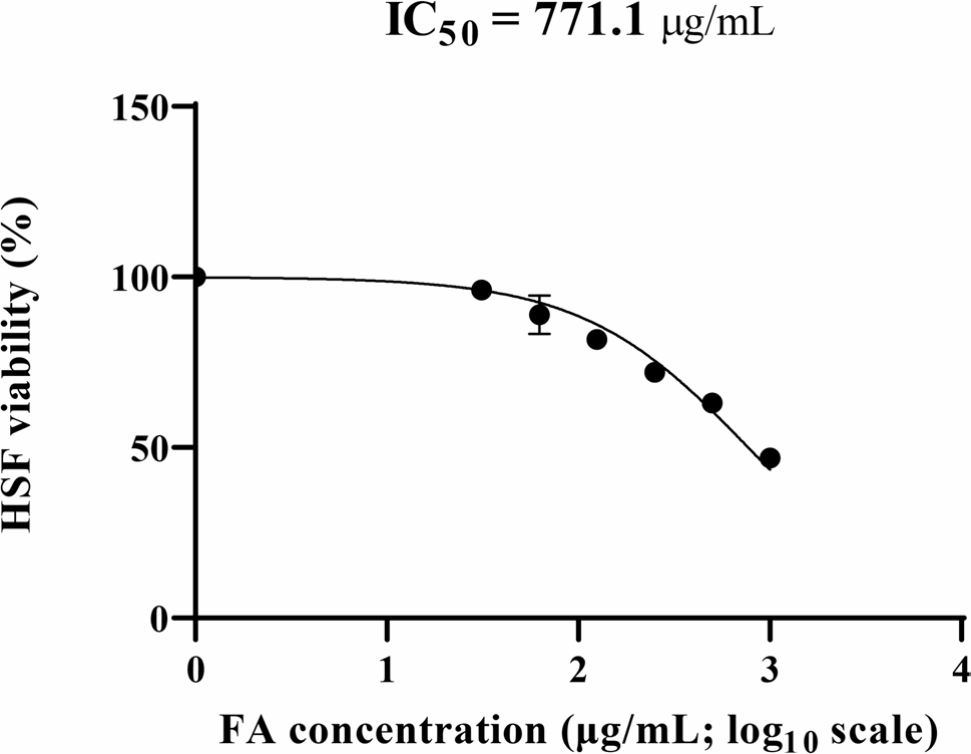
Dose-response curve illustrating the cytotoxic effect of ferulic acid (FA) on human skin fibroblast (HSF) normal cells using the MTT assay. The IC_50_ value of 771.1 µg/mL was derived from a non-linear regression fit of log_10_ concentration versus % viability. Data represent mean ± SD of three independent experiments

The cytotoxicity of FA was investigated on human skin fibroblast (HSF) normal cells using MTT assay. It was found that FA had an IC_50_ of 771.1 µg/mL, as shown in Fig. 12. The concentrations employed for topical in vivo treatment (512 µg/mL) and sub-MIC antibiofilm assays were at or below this value, supporting their suitability for localized applications, although systemic use is not implied.

### In vivo results

**Fig. 13:**
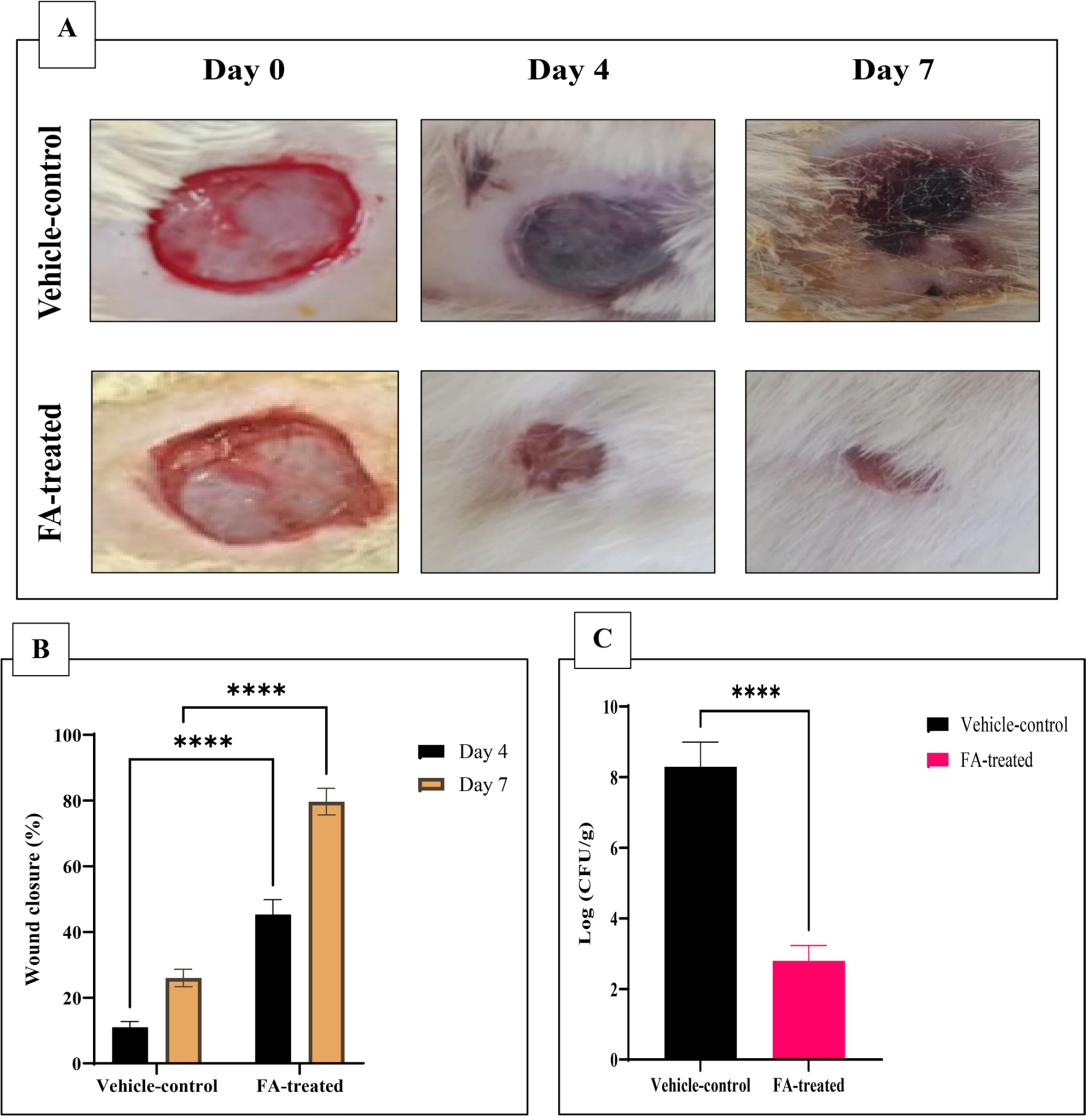
Therapeutic effect of ferulic acid (FA) on XDR *Acinetobacter baumannii* skin wound infection in rats. **A** Representative wound images from each group (vehicle-control vs. FA-treated) at designated time points. **B** Percentage of wound closure over time in vehicle-control and FA-treated groups. Data were analyzed by two-way ANOVA followed by Sidak’s post hoc test; ****p < 0.0001 (**C**) Mean log₁₀ bacterial load in 1 gram wound tissues from FA-treated and vehicle-control XDRAb-infected rats. Error bars represent standard deviations (*t*-test, ****p < 0.0001)

Administration of cyclophosphamide resulted in a significant reduction in neutrophil counts by approximately 65.5%, decreasing from 202.4 to 70 cells/mm³ in the treated animals. After the rats were infected with challenging *A. baumannii* (**Ac82**) topically, pharmacological therapy began 24 h after the infection daily along 6 days post-infection. The potential FA therapy against XDRAb infection in the rat wound model was assessed. FA improved the healing progress rate in the FA-treated (512 µg/mL + infected) animal groups compared to the vehicle-control (0.5% DMSO + infected) group (Fig. 13A). Results showed that a high percentage of wound closure occurred in the treated groups compared to the vehicle-control group (Fig. 13B). It was found that treating the infected wound with 512 µg/mL of FA resulted in approximately 82% wound closure on day 7 compared to the vehicle-control group, suggesting that the FA was effective in rapid healing rate. On the contrary, the open wound lesions of the vehicle-control animals were extremely inflamed with necrosis and presence of pus exudate indicating infection and unhealed wound.

**Fig. 14:**
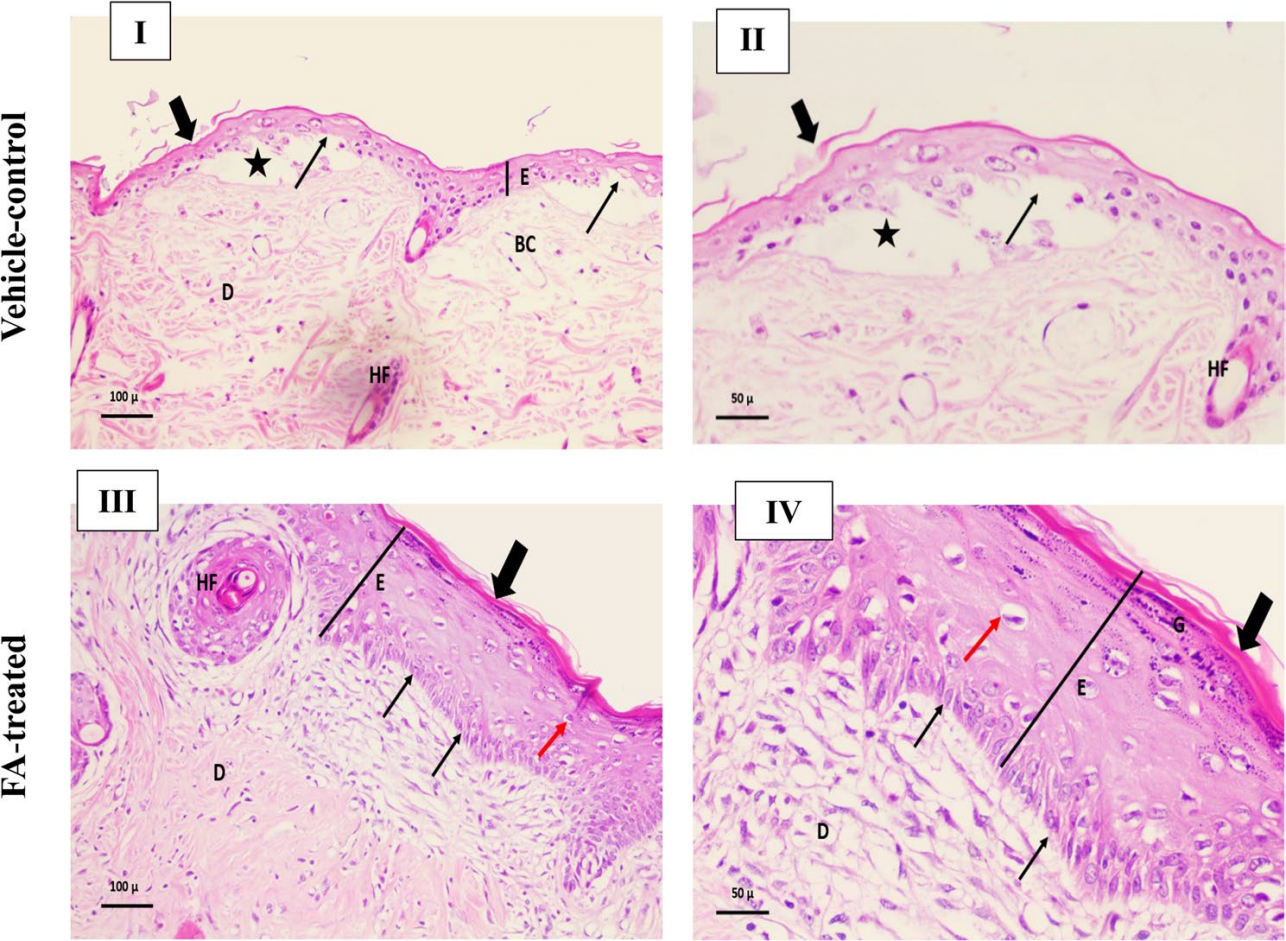
Representative hematoxylin and eosin (H&E)-stained sections demonstrating wound healing progression across experimental groups: (**I**) A photomicrograph of a section of skin wound of vehicle-control group exhibited impaired epidermal regeneration characterized by decreased epidermis thickness (E), thin stratum corneum with loss of its layers (thick arrow), disrupted basement membrane in certain areas (thin arrows), dermis of connective tissue (D) containing abundant inflammatory infiltrate with dense cellular accumulation with focal space (*), dilated blood capillary (BC) and appearance of hair follicle (HF), Magnification, x200. (**II**) A higher magnification of the vehicle-control group photomicrograph showing complete stratum corneum layer disruption (thick arrow), Subepidermal cleft formation (*), Basement membrane fragmentation (thin arrow) with marked keratinocyte depletion and appearance of hair follicle (HF). Absence of keratohyalin granules within epidermis, Magnification, x400 (**III**) A photomicrograph of a section of skin wound of ferulic acid-treated group displayed near-normal histoarchitecture characterized by normal thickness of epidermis (E), organized stratum corneum (thick arrow), intact basement membrane (thin arrows), normal keratinocytes except for vacuolation in few of them (red arrow), normal dermis of connective tissue (D) demonstrating reduced inflammatory infiltrate and better organized collagen deposition and hair follicle (HF), Magnification, x200 (**IV**) A higher magnification of the ferulic acid-treated group photomicrograph showing intact epidermal stratification (E), normal stratum corneum (thick arrow), reestablished keratohyalin granules (G), occasional keratinocyte vacuolation (red arrow), intact basement membrane (thin arrows) and preserved dermo-epidermal junction (D), Magnification, x400

The histopathological findings of skin wounds using H & E are presented in Fig. 14. The impact of FA on wound healing was evaluated. The vehicle-control group exhibited impaired wound healing, characterized by discontinuous surface squamous epithelium with prominent tissue gapping and wound bed filled with dense inflammatory cells infiltration (Fig. 14I**-**II). In contrast, wounds treated with FA (512 µg/mL) demonstrated significant histological improvement with successful re-epithelialization and restoration of the epidermal barrier. The epidermis showed well-organized stratified squamous epithelium with apparent wound closure. The dermal compartment exhibited reduced inflammatory cell infiltration compared to the vehicle-control group, with improved tissue organization. The dermis showed early signs of tissue remodeling with better structural arrangement of the extracellular matrix. Hair follicles remained well-preserved, indicating minimal collateral damage during the healing process (Fig. 14III-IV). These findings suggest that FA promotes accelerated wound repair by reducing inflammation, enhancing epithelial regeneration, and facilitating structured collagen deposition.

## Discussion

This study demonstrates that ferulic acid (FA) exerts dual antibacterial and antibiofilm effects against XDR *A. baumannii* (XDRAb) isolates recovered from cancer patients and that these effects translate into improved outcomes in a neutropenic wound infection model. These findings are particularly relevant in oncology and intensive care settings, where XDRAb has become a prominent cause of ventilator-associated pneumonia, bloodstream infections, and wound infections. In such environments, treatment-induced immunosuppression, extensive use of broad-spectrum antibiotics, and frequent invasive procedures converge to favor colonization by highly resistant, biofilm-forming strains [3, 4]. The 34% prevalence of XDRAb observed in our oncology cohort, together with near-universal resistance to β-lactams, fluoroquinolones, and aminoglycosides, and preserved susceptibility only to colistin, mirrors national and global reports describing severely constrained therapeutic options for this pathogen [6, 22, 48, 49].

The strong association between XDR status and high biofilm-forming capacity observed in our isolate collection underscores the dangerous synergy between genetic resistance and phenotypic tolerance. Biofilm formation on endotracheal tubes, wound surfaces, and indwelling devices enables *A. baumannii* to persist in hospital environments, restrict antibiotic penetration through an EPS-rich matrix, and generate physiological heterogeneity that promotes persister cell formation and chronic infection [50, 51]. Prior studies from Egypt and other high-burden regions have reported increasing prevalence of MDR and XDR *A. baumannii* with strong biofilm-forming capacity, often associated with poor outcomes in critically ill patients [22, 52, 53]. Our findings align with this epidemiological pattern and highlight the need for strategies that disrupt biofilm architecture and virulence programs rather than relying solely on bactericidal activity.

Several recent studies have investigated plant-derived compounds and nanoformulations as adjunctive approaches against multidrug- and colistin-resistant Gram-negative pathogens. However, most have focused on limited panels of *Enterobacteriaceae* or non-*Acinetobacter* species and emphasized restoration of antibiotic activity via *mcr-1* modulation or generalized ultrastructural damage [54]. Nano- and metal-based adjuvants, including cinnamon oil nanoemulsions and selenium or silver nanoparticles combined with conventional antibiotics, have been evaluated primarily against carbapenem- or colistin-resistant *Klebsiella pneumoniae*, *Pseudomonas aeruginosa*, or carbapenem-resistant *A. baumannii* [55–57]. In contrast, the present study systematically evaluates FA itself, rather than as a carrier or co-agent, against a well-defined collection of biofilm-forming XDRAb isolates from cancer patients, characterized by detailed antibiograms, MAR indices, and biofilm phenotypes, and supports FA as a dual-function topical candidate that exerts both direct antibacterial effects and robust antibiofilm activity without being combined with existing antimicrobials.​ Whereas another recent study for cinnamic acid derivatives, including FA, reported that they showed antibiofilm activity against MDR and colistin-resistant *A. baumannii*, but typically in small isolate sets and using limited assay panels relied mainly on MIC determination, crystal violet biofilm assays, membrane permeability testing, and SEM, without linking these effects to changes in cell surface properties, extracellular matrix composition, or biofilm-associated gene expression [12]. Our study extends this line of research through a multilayered analysis of a larger panel of clinically defined XDRAb isolates. We integrate phenotypic assays with transcriptional profiling of key biofilm-associated genes, complemented by in vivo validation in a neutropenic rat wound model.

The antibacterial activity of FA against our XDRAb isolates, with MICs of 512–1024 µg/mL, is comparable to reported values for cinnamic acids and polyphenols against MDR and colistin-resistant *A. baumannii* [12, 22]. Although these MICs exceed those of conventional antibiotics, the principal therapeutic value of FA in our model lies in its ability to disrupt biofilm formation and stability at sub-inhibitory concentrations. At ¼ MIC and ½ MIC, FA significantly (*p* < 0.05) inhibited biofilm formation and partially eradicated established biofilms while exerting minimal effects (*p* > 0.05) on planktonic growth kinetics. This profile is consistent with other plant-derived antivirulence agents that attenuate adhesion, quorum sensing, and matrix production without imposing strong bactericidal pressure, thereby potentially reducing selection for resistance [58–60].

Mechanistically, FA interferes with multiple stages of *A. baumannii* biofilm development. Treatment resulted in a concentration-dependent reduction in cell surface hydrophobicity, a key determinant of bacterial adhesion to abiotic surfaces and host tissues. This effect likely impairs initial attachment and colonization, consistent with known interactions of phenolic compounds with outer membrane proteins and surface-exposed structures, keeping cells in a planktonic state where they remain more accessible to host defenses and co-administered antibiotics [22, 53]. FA also significantly reduced EPS production, weakening the structural integrity of the biofilm matrix. These biochemical effects were corroborated by SEM and CLSM analyses, which revealed thinner, less cohesive biofilms with reduced matrix density and an increased proportion of damaged or non-viable cells compared to the untreated biofilm. MTT assays further demonstrated a substantial reduction in metabolic activity of biofilm-embedded bacteria at sub-MIC levels, indicating compromised biofilm viability. Together, these findings indicate that FA not only impairs the mechanical integrity of biofilms but also compromises the metabolic health of the remaining sessile cells.

At the molecular level, these phenotypic changes were accompanied by statistically significantly downregulated *abaI*, which encodes the N-acyl homoserine lactone synthase central to the *A. baumannii* quorum-sensing system, thereby likely dampening population-wide coordination of biofilm maturation and virulence factor expression. It also repressed *bfmR*, a response regulator that functions as a master switch for biofilm initiation and controls expression of the *csu* pili operon, as well as *csuE* itself, a structural component of the chaperone–usher pili essential for stable surface attachment. Downregulation of *bap*, which encodes a large surface protein that promotes intercellular adhesion, and *pgaB*, part of the *pgaABCD* locus responsible for synthesis and deacetylation of PNAG, a key EPS constituent, explains the formation of thinner, less cohesive biofilms with reduced matrix content under FA treatment. In contrast to reports showing that sub-MIC exposure to antibiotics such as colistin or imipenem may induce stress responses and biofilm-associated gene expression [61, 62], FA appears to shift the transcriptional program toward a less adherent, less matrix-rich, and less virulent phenotype.

An important strength of this study is the in vivo validation of FA in a neutropenic rat wound infection model using a clinically relevant, strong biofilm-forming XDRAb wound isolate. Chemotherapy-induced neutropenia and skin barrier disruption place cancer patients at high risk for persistent wound infections caused by XDRAb. In our model, cyclophosphamide-induced neutropenia allowed topical inoculation of the XDR isolate to establish a persistent infection, closely mimicking the clinical scenario. Topical application of FA at the MIC concentration (512 µg/mL) resulted in approximately 82% wound closure by day 7, a significant reduction in wound bacterial burden, and clear histological evidence of improved tissue repair compared with vehicle-treated controls (0.5% DMSO in the infected group), which exhibited extensive inflammation, necrosis, and persistent exudation, confirming that these effects were attributable to FA rather than the delivery vehicle. These findings align with previous work showing that FA has antioxidant and pro-healing properties, including the ability to scavenge reactive oxygen species and modulate growth factors in cutaneous wound models and in composite antioxidant hydrogels [63, 64]. The acceptable cytotoxicity profile observed in HSF, with an IC₅₀ above the MIC range used in our topical regimen, further supports the feasibility of developing FA-based wound formulations.Nevertheless, this study has limitations. It was conducted at a single center. Pharmacokinetic parameters and long-term safety were not assessed, and combination therapy with last-line antibiotics was not explored, despite previous demonstrations that FA can potentiate quinolone activity against *A. baumannii in vitro* and in systemic infection models, raising the possibility of synergistic or dose-sparing regimens [65]. In addition, we did not include a standard-of-care topical comparator (e.g. silver sulfadiazine) in the in vivo wound model, so direct benchmarking of FA against established therapies was not possible and should be addressed in future studies. Finally, the risk of resistance development to FA itself under prolonged exposure remains unknown and should be explored in future work. These limitations warrant further investigation.

## Conclusion

To the best of our knowledge, this work provides a comprehensive preclinical evaluation of FA as a multi-target antibiofilm agent against biofilm-forming XDRAb from cancer patients, linking mechanistic in vitro findings with in vivo efficacy in a clinically relevant wound infection model. By attenuating quorum sensing, adhesion, EPS production, and biofilm-associated gene expression at sub-MIC levels, FA exemplifies an antibiofilm-oriented strategy that may complement existing antimicrobial approaches. Future studies should focus on formulation optimization, pharmacological characterization, and evaluation of combination regimens in high-risk patient populations.

## Data Availability

No datasets were generated or analysed during the current study.
